# Pharmacological Targeting of DHHC9‐Mediated STRN4 Palmitoylation to Suppress YAP‐Driven Cancer Metastasis

**DOI:** 10.1111/jcmm.70815

**Published:** 2025-09-03

**Authors:** Yang Tian, Wei Li, Qing Zhai, Ying Yu, Jiaxin Yuan, Yan Ma, Jingjing Yang, Mingyue Li, Wenwen Chang, Wenjing Li, Keke Huang, Chongran Sun, Chen Zeng, Yingdi Sun, Jiabao Gu, Huilin Zhang, Dameng Li, Yanan Yu, Lu Hu, Peng Zhang, Bo Ma, Junnian Zheng, Pan Li, Feng Guo, Yang Sun

**Affiliations:** ^1^ Cancer Institute Xuzhou Medical University Xuzhou Jiangsu China; ^2^ Center of Clinical Oncology The Affiliated Hospital of Xuzhou Medical University Xuzhou China; ^3^ State Key Laboratory Jiangsu Center for the Collaboration and Innovation of Cancer Biotherapy, Cancer Institute Xuzhou Medical University Xuzhou Jiangsu China; ^4^ National Demonstration Center for Experimental Basic Medical Education Xuzhou Medical University Xuzhou Jiangsu China; ^5^ Jiangsu Province Key Laboratory of Immunity and Metabolism Xuzhou Medical University Xuzhou Jiangsu China; ^6^ Department of Pathogenic Biology and Immunology Xuzhou Medical University Xuzhou Jiangsu China; ^7^ Department of Genetics, School of Life Science Xuzhou Medical University Xuzhou Jiangsu China; ^8^ Cutaneous Biology Research Center, Massachusetts General Hospital Harvard Medical School Charlestown Massachusetts USA; ^9^ Department of Oncology, Tongji Medical College, Tongji Hospital Huazhong University of Science and Technology Wuhan Hubei China

**Keywords:** adenocarcinoma metastasis, hippo pathway, palmitoylation inhibitors, STRN4 palmitoylation, ZDHHC9

## Abstract

Protein S‐palmitoylation, a dynamic and reversible post‐translational modification involving the attachment of palmitate to cysteine residues, is a key regulator of protein functionality and cellular signalling. Dysregulation of this modification has emerged as a critical driver of cancer progression. Among the 23 DHHC palmitoyl transferases responsible for catalysing S‐palmitoylation, aberrant expression of specific members is linked to tumorigenesis and development, underscoring their potential as promising therapeutic targets. However, the cancer‐specific roles and substrates of individual DHHC enzymes remain poorly characterised. In this study, we identified DHHC9 as a crucial regulator of adenocarcinoma progression, including colorectal and lung cancers. Functional studies demonstrated that DHHC9 knockdown profoundly inhibited cell migration in vitro and tumour metastasis in vivo. Proteomic and functional analyses revealed that STRN4, a core component of the STRIPAK complex, was palmitoylated by DHHC9 at cysteine 701. The STRN4 palmitoylation reduced YAP phosphorylation, promoted nuclear translocation of YAP and activated downstream Hippo pathway transcriptional targets—including CCN1, CCN2 and ANKRD1—thereby driving cancer cell migration. Notably, we discovered two small molecules, Treprostinil and 10‐HCPT, as potent DHHC9 inhibitors that effectively suppressed adenocarcinoma cell migration. Our findings define the DHHC9‐STRN4‐YAP axis as a novel mechanism linking palmitoylation to phosphatase regulation and Hippo pathway dysregulation, unveiling DHHC9 as a highly promising therapeutic target in cancer treatment.

## 1 Introduction

The human proteome's diversity and functionality are intricately regulated by post‐translational modifications (PTMs), which are essential for cellular homeostasis and adaptation (PTMs) [1, 2]. Among these, S‐palmitoylation—a reversible lipid modification involving the covalent attachment of a palmitate molecule to specific cysteine residues—stands out due to its widespread occurrence and critical influence on protein activity, localisation and stability [3, 4, 5]. S‐palmitoylation has emerged as a key regulatory mechanism in various physiological and pathological processes, particularly in cancer progression, where it modulates the functions of oncogenes and signalling proteins, including PD‐L1, EGFR, NRAS and HRAS [6, 7]. The recent development of pharmacological agents targeting S‐palmitoylation underscores its promise as a therapeutic avenue, with candidates targeting proteins like STING and TEAD already in preclinical or clinical evaluation [8, 9, 10].

In the context of cancer, the enzymes responsible for mediating S‐palmitoylation have garnered increasing attention as potential therapeutic targets [6, 11]. Palmitoylation is catalysed by a family of 23 zinc finger Asp‐His‐His‐Cys (DHHC)‐type palmitoyl transferases (excluding DHHC10) [12, 13]. The aberrant expression of DHHC enzymes has been implicated in tumorigenesis and metastasis across various cancer types, with some acting as oncogenes and others as tumour suppressors [7]. For instance, DHHC3 is upregulated in metastatic breast cancer, and its loss reduces tumour size and metastasis and induces oxidative stress and senescence [14]. Similarly, DHHC20 is significantly implicated in pancreatic cancer through abnormal expression levels [15, 16]. Other DHHCs, such as DHHC11, DHHC12, DHHC15, DHHC22 and DHHC23, are aberrantly expressed in gliomas and are associated with poor outcomes [17]. Conversely, some DHHCs, such as DHHC1, exhibit tumour‐suppressive properties [18, 19], underscoring the context‐dependent roles of these enzymes in cancer biology.

Despite this progress, the precise roles of individual DHHC proteins in specific cancer types remain incompletely characterised. Notably, certain DHHC enzymes, such as DHHC8, exhibit dual roles depending on the cancer context, highlighting the complexity of their functions [20]. This underscores the necessity of elucidating the cancer‐specific roles of individual DHHCs. Furthermore, the identification of DHHC substrates is critical to understanding their functional contributions to cancer biology [21]. Proteomic studies have initiated valuable progress in mapping the substrates of DHHC3, DHHC7, DHHC11, DHHC15 and DHHC20, though opportunities remain for further exploration [22]. Additionally, the lack of comprehensive substrate mapping and selective inhibitors limits the therapeutic exploitation of this enzyme family. While broad‐spectrum inhibitors like 2‐BP have shown anticancer activity, their off‐target effects preclude clinical application [21]. This underscores the urgent need to unravel the cancer‐specific roles of DHHCs and identify their direct substrates to better understand their contributions to tumour progression.

To address these challenges, we aimed to systematically investigate the expression and functional roles of DHHC enzymes in cancer, focusing on identifying key drivers of tumour progression. Through integrative analysis, we identified DHHC9 as a critical regulator uniquely associated with adenocarcinomas, including colorectal, lung and pancreatic subtypes. Functional assays demonstrated that DHHC9 knockdown impairs adenocarcinoma cell migration both in vitro and in vivo. Using quantitative proteomics, we uncovered STRN4, a key component of the STRIPAK complex, as a novel DHHC9 substrate. Loss of STRN4 palmitoylation disrupted YAP nuclear localisation and suppressed the expression of Hippo pathway targets, thereby inhibiting cancer cell migration. Finally, we identified two promising DHHC9 inhibitors, treprostinil and 10‐HCPT, which effectively suppressed adenocarcinoma metastasis in models. Taken together, our findings establish DHHC9 as a pivotal regulator of adenocarcinoma progression, a kind of cancer with hyperactivation of YAP/TAZ. Our results also advance palmitoylation as a druggable regulator of phosphatase activity and provide a roadmap for targeting the ‘undruggable’ Hippo pathway in metastatic cancers.

## 2 Materials and Methods

### 2.1 Reagents

**Table:** 

Reagents	Vendor	Cat no.
100 bp DNA Ladder	Vazyme, China	MD104‐01
10‐Hydroxycampto	MCE, U.S.	HY‐N0095
180 kDa Prestained Protein Marker	Vazyme, China	MP102‐02
Acetonitrile LC–MS grade	Aladdin, China	A120771
AgeI‐HF	New England Biolabs, U.S.	R3552S
Ampicilin	Beyotime, China	ST007‐5 g
Anti‐DDDDK‐tag mAb	MBL, Japan	M185
Anti‐HA‐tag mAb	MBL, Japan	M180‐3
Biotin HPDP	Meilunbio, China	MB5215
Biotin‐azide	CONFLUORE, China	BBBE‐31
ChamQ SYBR Colour qPCR Master Mix	Vazyme, China	Q312
Copper sulfate, pentahydrate	Aladdin, China	C112396
Crystal violet staining solution	Innochem, China	a47220
D•FBS Premium	PAN, Germany	ST30‐2102
DAPI	Beyotime, China	C1002
DH5α	In‐house made	
DMEM	Thermo Fisher, U.S.	12,100,046
DNA ladder (1 kb)	Abclonal, China	RM19005
dNTP mix	New England Biolabs, U.S.	R0176L
Doxycycline	MCE, U.S.	HY‐N0565
DpnI	New England Biolabs, U.S.	R0176S
DTDP	Sigma, U.S.	D5767
EcoRI‐HF	New England Biolabs, U.S.	R3101V
EndoFree Maxi Plasmid Kit	TIANGEN, China	DP117
EndoFree Mini Plasmid Kit II	TIANGEN, China	DP118‐02
Enhanced BCA Protein Assay Kit	Beyotime, China	P0009
Estriol	MCE, U.S.	HY‐B0412
FastPure Gel DNA Extraction Mini Kit	Vazyme, China	DC301‐01
Fatty acid–free BSA	Sigma, U.S.	SRE0098
FBS Premium	PAN, Germany	ST30‐3302
Formamide	Innochem, China	1975/12/7
Formate LC–MS grade	TCI, China	F0654
Ganti‐Rabbit Secondary Antibody Alexa Fluor 568	Thermo Fisher, U.S.	2,379,475
GAPDH Mouse mAb (high dilution)	Abclonal, China	AC033
Goat anti‐Rabbit IgG (H + L)	Thermo Fisher, U.S.	31,460
Haematoxylin and Eosin staining	Yuanye, China	R20570
Haematoxylin blue return solution	Servicebio, China	G1040
Haematoxylin differentiation solution	Servicebio, China	G1039
HiScript III All‐in‐one RT SuperMix Perfect	Vazyme, China	RT101
HRP Goat Anti‐Mouse IgG (H + L)	Abclonal, China	AS003
Iodoacetamide	Aladdin, China	I131590
Isoleojaponin	MCE, U.S.	HY‐19928
Isopropyl Alcohol	Sinopharm Chemical, China	40,064,360
Methyl alcohol	Sinopharm Chemical, China	10,014,108
Mouse (E5Y6Q) mAb IgG2a Isotype Control	CST, U.S.	61656S
MTT	Solarbio, China	M8180
NEM	Aladdin, China	E100553
Neobavaisoflavone	MCE, U.S.	HY‐N0720
N‐Methylhydroxylamine hydrochloride	Sigma, U.S.	255,580
PEI	Polysciences, U.S.	24,765–1
Phosphatase Inhibitor Cocktail	APExBIO, U.S.	K1012
Phospho‐YAP1‐S128 Rabbit pAb	Abclonal, China	AP1187
Phusion High‐Fidelity DNA Polymerase (2 U/μL)	Thermo Fisher, U.S.	F530S
Poly‐D‐lysine	Beyotime, China	ST508
Polyethylene glycol	Aladdin, China	P103737
Protease Inhibitor Cocktail	APExBIO, U.S.	K1007
Protein A + G Agarose (Fast Flow, for IP)	Beyotime, China	P2055
PVDF membrane	Cytiva (GE), U.S.	10,600,023
Q5 high fidelity DNA polymerase	New England Biolabs, U.S.	M0491L
RNAiso Plus	Takara, Japan	9109
RPMI Medium 1640	Thermo Fisher, U.S.	31,800–022
Stb13	In‐house made	
Streptavidin Agarose	Beyotime, China	P2159
T4 DNA Ligase	New England Biolabs, U.S.	M0202S
T4 Polynucleotide Kinase	New England Biolabs, U.S.	M0201L
TBTA	Aladdin, China	T162437
TCEP	Sigma, U.S.	C4706
Treprostinil	Macklin, China	T854794
Trichloromethane	Sinopharm Chemical, China	10,006,891
Trypsin	Beijing Shengxia Prot, China	LP0042B
Ultra GelRed (10,000×)	Vazyme, China	GR501‐01
YAP1 Polyclonal antibody	Proteintec, China	13,584–1‐AP
ZipTip with 0.6 μL C18 resin	Millipore, U.S.	ZTC18S096
β‐Actin Rabbit mAb (High Dilution)	Abclonal, China	AC026
pcDNA3.1‐DHHC9‐3 × HA	Youbio, China	
pcDNA3.1‐STRN4‐3 × Flag	Youbio, China	
Tet‐pLKO‐puro	Kept in lab	
pLKO.1‐TRC cloning vector	Kept in lab	
psPAX2	Kept in lab	
pCMV‐VSV‐G	Kept in lab	

### 2.2 Database and Bioinformatic Analysis of the Biological Significance of ZDHHCs in Pan‐Cancer

To explore the biological significance of DHHC palmitoyl transferases across various cancer types, we performed a comprehensive bioinformatic analysis using publicly available datasets from *The Cancer Genome Atlas* (TCGA). The following analyses were conducted to assess mutation frequency, copy number variation (CNV), differential expression and prognostic relevance of DHHC family members in pan‐cancer.

Data Acquisition and Processing:

RNA‐sequencing (RNA‐seq) expression data, mutation profiles and CNV data for 33 cancer types were retrieved from the TCGA database via the Genomic Data Commons (GDC) portal (https://portal.gdc.cancer.gov/). All expression data were normalised using the fragments per kilobase of transcript per million mapped reads (FPKM) method. Clinical annotations, including overall survival (OS) and disease‐free survival (DFS) data, were also extracted for downstream analysis.

### 2.3 Mutation and Copy Number Variation Analysis

The mutation frequency and types of alterations (e.g., single nucleotide variants and insertions/deletions) in DHHC genes were analysed using the *Maftools* package in R. CNV analysis was conducted using GISTIC 2.0 to identify recurrent amplifications and deletions across the pan‐cancer cohort. The statistical significance of copy number alterations was assessed, and results were visualised using heatmaps and oncoplots.

### 2.4 Differential Gene Expression Analysis

Differential expression analysis of DHHC family members between tumour and adjacent normal tissues was conducted using the *edgeR* package in R. A threshold of |log2 fold change| > 1 and an adjusted *p*‐value < 0.05 were applied to identify significantly dysregulated genes. Gene expression patterns were visualised using volcano plots and heatmaps.

### 2.5 Unsupervised Clustering and Principal Component Analysis (PCA)

Unsupervised hierarchical clustering was performed to categorise tumours based on DHHC expression profiles using the *ConsensusClusterPlus* package in R. Principal Component Analysis (PCA) was conducted using the *Limma* package in R to visualise clustering patterns and identify potential cancer subtypes associated with specific DHHC expression signatures.

### 2.6 Survival Analysis

The prognostic value of DHHC genes was evaluated using Kaplan–Meier survival analysis in https://kmplot.com/analysis, with indicated instruction [23, 24].

### 2.7 Cell Culture

Human HEK293T, HCT116, DLD‐1, A549 and mouse CT‐26 cells were obtained from the National Collection of Authenticated Cell Cultures (NCACC) and the National Infrastructure of Cell Line Resource (NICR). HEK293T, HCT116 and A549 were cultured in Dulbecco's modified Eagles medium (DMEM) (Life Technologies) supplemented with 10% FBS (PAN‐Biotech GmbH) and 100 units/mL penicillin and 100 μg/mL streptomycin; DLD‐1 and mouse CT‐26 were cultured in the Roswell Park Memorial Institute 1640 Medium (RPMI‐1640) (Life Technologies) supplemented with 10% FBS (PAN‐Biotech GmbH) and 100 units/mL penicillin and 100 μg/mL streptomycin. All cell lines are free of mycoplasma contamination. All cell lines used in this study were authenticated by identification of short tandem repeat (STR) markers.

### 2.8 Transfection

Plasmid transfection was performed using PEI (1 μg/μL). Briefly, cells were seeded 1 day prior to transfection. The DNA was diluted in serum‐free MEM and mixed with PEI at a DNA:PEI ratio of 1:3. After incubating the mixture at room temperature for 20–30 min, it was added directly to the cells. Gene expression levels were assessed 48 h post‐transfection.

### 2.9 Lentiviral Production and Generation of DHHC9 Knockdown Cell Lines

To generate HCT116, A549 and DLD‐1 shDHHC9 knockdown cell lines, shRNAs targeting DHHC9 were cloned into the Tet‐pLKO‐puro vector. HEK293T cells were seeded in a 10 cm dish and grown to 80% confluence before being transfected with 10 μg of the transfer plasmid, 5 μg of shRNA, 3 μg of psPAX2, 2 μg of VSV‐G and 30 μL of PEI transfection reagent in serum‐free MEM. The culture medium was replaced after overnight incubation. After 48 h, viral supernatants were collected, filtered through a 0.45 μm low‐protein‐binding membrane (Millipore) and supplemented with polybrene before being used to infect target cells. Transduced cells were subsequently selected with puromycin for 1 week.

The shRNA sequences of human DHHC9 were as follows:

sh*DHHC9*‐1: GAGGAACTACCGCTACTTCTA.

sh*DHHC9*‐2: GAAGTCCTCATTTGCTTCTTT.

For CT‐26 cells, shRNAs of sh*Dhhc9* were cloned into pLKO.1‐TRC cloning vector. The lentiviral production and stable cell line generation procedure were the same as those for human cell lines. The shRNA sequences of mouse *Dhhc9* and control were as follows:

sh*Dhhc9*‐1: CAACCAGATTGTGAAACTGAA.

sh*Dhhc9*‐2: CGCCTTTAACATCGTCTATGT.

shControl: CCTAAGGTTAAGTCGCCCTCG.

### 2.10 DNA Constructs and Mutagenesis

DHHC9‐3 × HA and STRN4‐3 × Flag plasmids were purchased from Youbio. Site‐directed mutagenesis was performed using the QuikChange method (Agilent Technologies) to generate the DHHC9‐3 × HA C169S and STRN4‐3 × Flag C701S mutants. The oligonucleotide primers used for mutagenesis are listed below:

Human DHHC9–3 × HA C169S: ACCCAGGGGGAGTGATGGTCGAAGCGC.

Human STRN4‐Flag C701S: AGCCATGACTCCTCCCTGCGT.

### 2.11 Metabolic Labeling, Click Reaction and Streptavidin Pulldown

Metabolic labeling, click reaction and streptavidin pulldown were performed according to the published procedure with slight modifications [9, 25]. Briefly, HEK293T cells were incubated overnight in a medium supplemented with 10% fatty acid–free FBS, with either DMSO or the probe (Alkynyl Palmitic acid) added for labeling. The cells were lysed with a lysis buffer containing 50 mM HEPES (pH 7.4), 150 mM NaCl, 1% NP‐40 and EDTA‐free protease inhibitors followed by Click reaction with biotin‐Azide. Then methanol, chloroform and water were added in a sequential volume ratio of 4:1:3:1 to precipitate the proteins. The precipitated proteins were then collected by centrifugation at 17,000 × g for 15 min, resuspended in a suspension buffer composed of PBS, 0.05% Tween‐20 and 2% SDS and diluted with PBST (PBS with 0.05% Tween‐20), and the labelled cellular proteins were enriched using streptavidin agarose (Beyotime, China) at room temperature with rotation for 2 h. After washing with PBST four times, bound proteins were eluted with elution buffer (6× SDS‐PAGE sample buffer diluted with 10 mM EDTA pH 8.2 and 95% formamide) and boiled at 95°C for 10 min. Samples were separated by SDS‐PAGE and analysed by immunoblotting.

### 2.12 Acyl‐Biotin Exchange (ABE) Assay

HEK293T cells were transfected with the STRN4‐3xFlag expression construct vector. After 48 h, the cells were collected, washed three times with PBS, lysed with lysis buffer (2% SDS, w/v, 150 mM NaCl, 5 mM EDTA, pH 7.4) and sonicated. Cell debris was removed by centrifugation at 17,000 × g for 15 min at room temperature. Protein concentration was determined by the BCA method (Beyotime, China). 1 mg (5 mg/mL) protein was used for the ABE assay according to the published procedure with slight modifications [26, 27]. TCEP was added to the supernatant to reduce the disulfide bonds. *N*‐ethylmaleimide (NEM) and DTDP were used to block free sulfhydryl groups on the proteins. Then the samples were treated either with or without 2 M hydroxylamine (HAM) and 2 mM biotin‐HPDP to label the palmitoylation sites. S‐acylated proteins were enriched by streptavidin agarose and finally analysed by SDS‐PAGE.

### 2.13 Immunoprecipitation and Immunoblotting

Cultured cells were lysed with a modified lysis buffer (50 mM HEPES pH 7.4, 150 mM NaCl, 1% NP‐40) supplemented with a protease and phosphatase inhibitor cocktail. Cell debris was removed by centrifugation at 17,000 × g for 15 min at 4°C. The supernatants (3 mg protein/mL) were incubated with the specific antibodies overnight and then mixed with protein A/G agarose beads (Beyotime) for 8 h at 4°C. Immunocomplexes were washed with PBS four times and were subjected to immunoblot analysis with corresponding primary and HRP‐conjugated secondary antibodies. Immunoblots were visualised using the chemiluminescent substrate, and the band intensity was quantified by the Image Lab software program (Bio‐Rad Laboratories, CA).

Antibody and dilutions used in the studies: Anti‐DDDDK‐tag mAb (MBL, Cat no. M185‐3 L, 1:5000), Anti‐HA‐tag mAb (MBL, Cat no. M180‐3 1:5000), β‐Actin rabbit mAb (Abclonal, Cat no. AC026, 1:10000), GAPDH (Abclonal, Cat no. AC033, 1:50000), YAP1 (Proteintect, China, Cat no. 13584–1‐AP, 1: 1000), p‐YAP (S128) (Abclonal, Cat no. AP1187, 1: 1000), p‐YAP (S127) (Proteintech, Cat no.), Anti‐rabbit HRP (Thermo Fisher, Cat no. 31460, 1:5000), Anti‐mouse HRP (Abclonal, Cat no. AS003, 1:5000).

### 2.14 Cell Viability Assay

Cell viability was assessed using the MTT assay. Doxycycline‐induced DHHC9 knockdown HCT116, A549 and DLD‐1 cells were seeded into 96‐well plates at a density of 2000 cells per well. After 12 h, the culture medium was replaced with fresh medium containing either doxycycline or vehicle control. At the indicated time points, MTT solution (5 mg/mL) was added to each well and incubated for 2 h at 37°C. Subsequently, 100 μL of DMSO was added to dissolve the formazan crystals formed. Cell viability was measured by recording absorbance at 595 nm using a microplate reader. The viability of untreated cells at day 0 was set to 100%, and the relative viability of doxycycline‐treated cells was calculated accordingly.

### 2.15 Scratch Wound Healing Assays

HCT116, A549 and DLD‐1 cells were pre‐treated with doxycycline for 24 h to induce DHHC9 shRNA expression, then seeded into six‐well plates and incubated overnight at 37°C. The following day, once the cells reached 100% confluence, a scratch was made across the monolayer using a 200‐μL pipette tip. After washing twice with PBS, the cells were treated with serum‐free DMEM containing either DMSO or doxycycline. Images of the scratch area were captured at 0 h (before treatment), 24 h and 48 h post‐incubation using a microscope equipped with a built‐in camera at 10× magnification. Wound closure (%) was calculated using the following equation:

$$\text{Wound area}\;\left(\%\right)=\frac{\text{Area between cells}\;\mathrm{at}\;0\;h–\text{Area between cells}\;\mathrm{at}\;24\;\text{or}\;48\;h}{\text{Area between cells}\;\mathrm{at}\;0\;h}$$

### 2.16 Transwell Migration Assay

HCT116, A549 and DLD‐1 cells were pre‐treated with doxycycline for 24 h to induce DHHC9 shRNA expression, then seeded into 24‐well transwell chambers equipped with an 8.0 μm pore polycarbonate filter (Falcon, BD, Cat no. 353097). The chambers were incubated at 37°C with 5% CO₂ for 24 h. Following incubation, the upper surface of the filters was gently scraped with cotton swabs to remove non‐migrating cells. The remaining cells on the lower side of the membrane were fixed with 4% paraformaldehyde and stained with 0.1% crystal violet. The migration area was quantified in five high‐power fields per filter using ImageJ software.

### 2.17 Immunofluorescent Staining and Confocal Microscope

Cells were seeded on glass coverslips in six‐well plates. After 48 h of doxycycline induction, the cells were washed three times with PBS and fixed with 4% paraformaldehyde for 15 min at room temperature. Following fixation, the cells were washed three more times with PBS and permeabilised with 0.1% Triton X‐100 for 5 min, followed by another three PBS washes. The cells were then blocked with blocking buffer (5% BSA in PBS) for 1 h and incubated overnight at 4°C with a YAP antibody at a 1:1000 dilution. The next day, the slides were incubated for 1 h with Alexa Fluor 568‐conjugated secondary antibody (Goat anti‐Rabbit IgG (H + L), Cross‐Adsorbed Secondary Antibody, Thermo Fisher, Cat no. 2379475) at a 1:1000 dilution in blocking buffer. Finally, the coverslips were mounted using mounting media containing 1 μg/mL DAPI (Beyotime, Cat no. C1002) for nuclear staining. Images were captured using an LSM880 inverted confocal microscope equipped with a ×63 objective lens (Zeiss, NY). Image analysis and manual quantification were performed using ZEN Blue software.

### 2.18 Quantitative RT‐PCR

Total RNA was extracted using TRIzol reagent according to the manufacturer's instructions, and cDNA was synthesised using the HiScript III All‐in‐One RT SuperMix Perfect for qPCR (Vazyme, Cat no. RT101) with random primers. Quantitative PCR was performed using the ChamQ SYBR Colour qPCR Master Mix (Vazyme, Cat no. Q312) on a Roche LightCycler 96 system. Gene expression levels were normalised to β‐actin, which was used as an endogenous control to calculate relative expression data.

Primer sequences are as follows: *DHHC9* forward, CTTTGAGTGCCGCTACCTG; *DHHC9* reverse, ACTGAAGCTGGTCCTCAACA; *CCN1* forward, GGAAAAGGCAGCTCACTGAAGC; *CCN1* reverse, GGAGATACCAGTTCCACAGGTC; *CCN2* forward, CTTGCGAAGCTGACCTGGAAGA; *CCN2* reverse, CCGTCGGTACATACTCCACAGA; *ANKRD1* forward, CGACTCCTGATTATGTATGGCGC; *ANKRD1* reverse, GCTTTGGTTCCATTCTGCCAGTG; *ACTB* forward, CACCATTGGCAATGAGCGGTTC; *ACTB* reverse, AGGTCTTTGCGGATGTCCACGT; *Dhhc9* forward, CTGCTGTGAAGTGCTTTGTGGC; *Dhhc9* reverse, TCTGTGGCAACAGGCTACTGCT; *Actb* forward, CATTGCTGACAGGATGCAGAAGG; *Actb* reverse, TGCTGGAAGGTGGACAGTGAGG.

### 2.19 Sample Preparation for S‐Palmitoylated Proteome Analysis

HCT116, A549 and DLD‐1 cells were pre‐treated with doxycycline for 24 h to induce DHHC9 shRNA expression. Cells were then collected and lysed as described in the ABE assay method above. A total of 3 mg of protein was used for S‐palmitoylated proteome analysis. TCEP was applied to reduce disulfide bonds, and all free thiol groups were irreversibly blocked using NEM and DTDP. The protein samples were then equally divided into two portions: one treated with neutral hydroxylamine (HA) to specifically cleave off S‐acyl moieties, and the other left untreated as a control. Following this, newly formed thiol groups were labelled with biotin‐HPDP. After completing the ABE chemical reactions, whole protein lysates were digested in solution, and biotinylated peptides—representing previously S‐acylated proteins—were selectively enriched using streptavidin affinity agarose. Non‐acylated peptides, potentially originating from both S‐acylated and non‐acylated proteins, were thoroughly washed away. Bound peptides were gently eluted using TCEP, restoring the originally S‐acylated cysteine residues to free cysteines and subsequently analysed by LC–MS/MS.

### 2.20 Nano‐LC–MS/MS Analysis and Data Analysis

Digestion solution was acidified by 5% formic acid, and peptides were desalted prior to LC–MS/MS analysis using in‐house C18 Ziptips (Merk Millipore, cat. ZTC18S096). Peptide samples were loaded onto a 20 cm long, 75 μm I.D fused silica capillary columns packed with Reprosil‐Pur C18‐AQ resin (3 mm; Dr. Maisch GmbH, Germany) and resolved by an EASY‐nLC 1000 HPLC system (Thermo Fisher Scientific), coupled in‐line with a Q‐Exactive HF (Thermo Fisher Scientific). The HPLC gradient was 2%–30% solvent B (A = 0.1% formic acid in water; B = 0.1% formic acid in acetonitrile) for 70 min, followed by 30%–95% solvent B for 10 min, and then held at 95% solvent B for 10 min, with a constant flow rate of 300 nL/min. Full MS spectrum scans (m/z 350–1600) were performed at a resolution of 70,000 (at 200 m/z), and the three most intense ions were selected for MS/MS performed with high‐energy collision dissociation (HCD) with normalised collision energy of 25 at a resolution of 17,500 (at 200 m/z). AGC targets of full MS and MS/MS scans were 13,106 and 53,104, respectively. Unassigned charge states and singly charged species were rejected; dynamic exclusion was set to 30 s.

Database searching and label‐free quantification were based on MaxQuant software. Two trypsin miss‐cleavage sites were allowed, and precursor ion and fragment ion tolerances were set to 10 ppm and 0.02 Da, respectively. Oxidation (+15.9949) on methionine and N‐ethylmaleimide (125.0477) on cysteine were set as dynamic modifications. A peptide score of 20 was chosen to filter the peptide identification matches.

### 2.21 Proteomic Data Analysis and Bioinformatics

A widely used label‐free quantitative method in MaxQuant software was applied for protein quantification. To distinguish S‐acylated proteins from contaminants, the Q value was calculated between +HA and −HA groups in both *DHHC* knockdown (+Dox, with doxycycline) and control (−Dox, without doxycycline) samples. Proteins with Q values < 0.1 were accepted as *S*‐acylated protein candidates. Statistical analysis for chemical genetic DHHC9 substrates for cellular compartment and biological process GO terms compared to the human proteome showed terms with Fold change < 0.5 (+Dox vs. −Dox) and −Log10(p‐value) > 2 (+Dox vs. −Dox) from an FDR adjusted Fisher's exact test for HCT116 cells. As for A549 cells, Fold change < 0.5 (+Dox vs. −Dox) and −Log10(p‐value) > 2 (+Dox vs. −Dox) were set.

### 2.22 Protein–Protein Interaction Network Analysis

STRING (Search Tool for the Retrieval of Interacting Genes/proteins, https://string‐db.org/) is designed to collect, score and integrate all public sources of protein–protein interaction (PPI) information, and further calculations are used to construct PPI networks and predict potential interactions [28] In our research, we analysed the interaction among the potential DHHC9 substrates shared by HCT116 and A549 cells in S‐palmitoylation proteomics, and the STRING interaction network was laid out by stringApp in Cytoscape 3.4.0 [29].

### 2.23 Thermal Shift Assay

DHHC9‐3 × HA plasmids were transfected into HCT116 cells and allowed to express for 48 h. The thermal shift assay of cell lysates was performed as described in previous literature. Briefly, cultured HCT116 cells were lysed in 0.2% NP‐40 lysis buffer supplemented with a protease and phosphatase inhibitor cocktail through repetitive freeze–thaw cycles. The resulting supernatant was divided into two aliquots: one treated with treprostinil and the other with the solvent control. After a 60‐min incubation, each sample was further divided into six aliquots and subjected to heating at designated temperatures for 3 min using a 96‐well thermal cycler, followed by cooling to room temperature for 3 min. The lysates were then centrifuged at 17,000 × g for 20 min at 4°C, and the supernatant was analysed by immunoblotting for target proteins.

### 2.24 Mouse Model and Tumour Studies

All animal procedures were performed in accordance with the guidelines of the institutional animal care and use committee of Xuzhou Medical University. Ethical approval for research involving animals has been obtained (IACUC Issue, No. 202210S029). Mouse Room Conditions: light cycle: 12 light/12 dark cycle was used; temperature: 18°C–23°C; humidity: 40%–60%.

To establish the spleen‐to‐liver metastasis model, mice were anaesthetised and received an intra‐splenic injection of CT‐26 murine colorectal carcinoma cells (5 × 10^5^ cells in 20 μL PBS) using insulin syringes. Following surgery, mice were monitored daily for recovery, with wound clips removed on day 7, and body weights recorded every other day. All mice were sacrificed 14 days post‐injection for tissue collection (liver and spleen).

To assess the effect of DHHC9 knockdown on metastasis, mice were randomly divided into three groups (*n* = 7–10 per group) upon cell injection: one group received control shRNA‐transduced CT‐26 cells (*shCon*), while the other two groups received CT‐26 cells transduced with distinct shRNAs targeting DHHC9 (*shDhhc9#1* and *shDhhc9#2*).

To evaluate the impact of pharmacological agents on metastasis, mice were randomly assigned to treatment groups upon cell injection (*n* = 5–8 per group). Groups received systemic administration of either vehicle control, Treprostinil (100 μg/kg body weight via i.p.) or 10‐Hydroxycamptothecin (10‐HCPT; 2 mg/kg body weight via i.p.). Treatments commenced on day 3 post‐surgery and were administered daily until the experimental endpoint. At sacrifice, liver and spleen tissues were harvested at sacrifice for subsequent analysis.

For the Orthotopic mouse model of Colorectal Cancer, BALB/c male mice (18–20 g), at age 8–10 weeks, were purchased from Jicui (GemPharmatech Co. Ltd., China). The mice were randomly assigned to three groups, with 5 mice in each group. As reported previously, 1 × 10^6^ CT—26 cells suspended in 15 μL of PBS were injected between the cecum and rectum of the mice using insulin syringes (BD Biosciences). Throughout the following weeks, the animals were monitored daily to ensure they were recovering well. The surgical staples were removed 7 days post‐operation. The body weights of the mice were recorded every other day. After 25 days, the experiment was terminated, and the spleen and colon of each mouse were harvested.

### 2.25 Haematoxylin and Eosin Staining

Mice autopsy was conducted to examine distant organ metastatic tumour nodules. Tumour samples were collected for histological evidence of the tumour phenotype. Histological examination: Liver tissues were fixed in 4% paraformaldehyde, embedded in paraffin for haematoxylin and eosin (H&E) staining, which was performed according to the standard procedures.

### 2.26 RNA‐Seq

HCT116 cells were treated with doxycycline for 48 h to induce sh*DHHC9* mRNA expression. Total RNA was isolated with Trizol reagent (Invitrogen). The integrity of isolated RNA was analysed using an Agilent 2200 Bioanalyser, and the cDNA libraries were constructed for each RNA sample using the VAHTS Universal V6 RNA‐seq Library Prep Kit for Illumina (vazyme Inc.). The libraries were quality controlled with Agilent 2200 and sequenced by DNBSEQ‐T7 on a 150 bp paired‐end run. Before read mapping, clean reads were obtained from the raw reads by removing the adaptor sequences and low‐quality reads. The clean reads were then aligned to the human genome (GRCh38, Ensembl104) using Star. HTseq was used to get gene counts.

The different expression genes were selected with a log‐fold expression change of log2FC > 1 or < −1 and a threshold of false discovery rates (FDR < 0.05). DEGs were filtered using a DEGSeq algorithm in R.

Gene ontology (GO) was applied to analyse the main functions of DEGs according to the GO database and to determine the biological implications of unique genes in the significant or representative profiles.

The identification of significant pathways for DEGs was according to the Kyoto Encyclopedia of Genes and Genomes (KEGG) database5. A Fisher exact test was used to screen the significant enrichment pathways with a threshold of significance defined by P‐value and FDR. The top 10 pathway categories with FDRs < 0.05 were reported.

Gene Set enrichment analysis (GSEA) was performed in R, and the YAP/TAZ‐TEAD molecular signatures database was used according to the previously published report [30, 31].

### 2.27 Drug Virtual Screening

The crystal structure of the DHHC9 protein (PDB ID: 8HF3, Chain A) was retrieved from the RCSB PDB database and prepared using the Protein Preparation Wizard module in Schrödinger software, involving preprocessing, native ligand state regeneration, hydrogen bond optimisation, energy minimisation and water molecule removal. Ligands from eight compound libraries—Selleck FDA‐Approved Drug Library (L1300), TargetMol Natural Compound Library (L6000), MedChemExpress Natural Product Library (HY‐L021), Pharmacodia Natural Product Library, BioBioPha BBP Natural Product Library, APExBIO Anticancer Compound Library Plus (L1023), Selleck Preclinical/Clinical Compound Library (L2000) and MedChemExpress Immunology/Inflammation Compound Library (HY‐L007)—were processed using the LigPrep module to generate 3D chiral conformations. ADME/T properties of the ligands were predicted using the QikProp module, evaluating 51 parameters with reference to Lipinski's Rule of Five. Active sites of DHHC9 were identified using the SiteMap module, and receptor grids were generated with the Receptor Grid Generation module to define the binding pocket. Molecular docking was performed with HTVS, SP and XP modes, progressively increasing docking precision, and docking scores were recorded to assess binding free energy and stability. Finally, MM‐GBSA analysis was conducted on the lowest‐scoring ligands to approximate binding free energy (dG Bind), with lower values indicating higher stability between the ligands and DHHC9.

## 3 Results

### 3.1 Identification of *DHHC9* as a Potential Key Regulator in Adenocarcinoma

**FIGURE 1:**
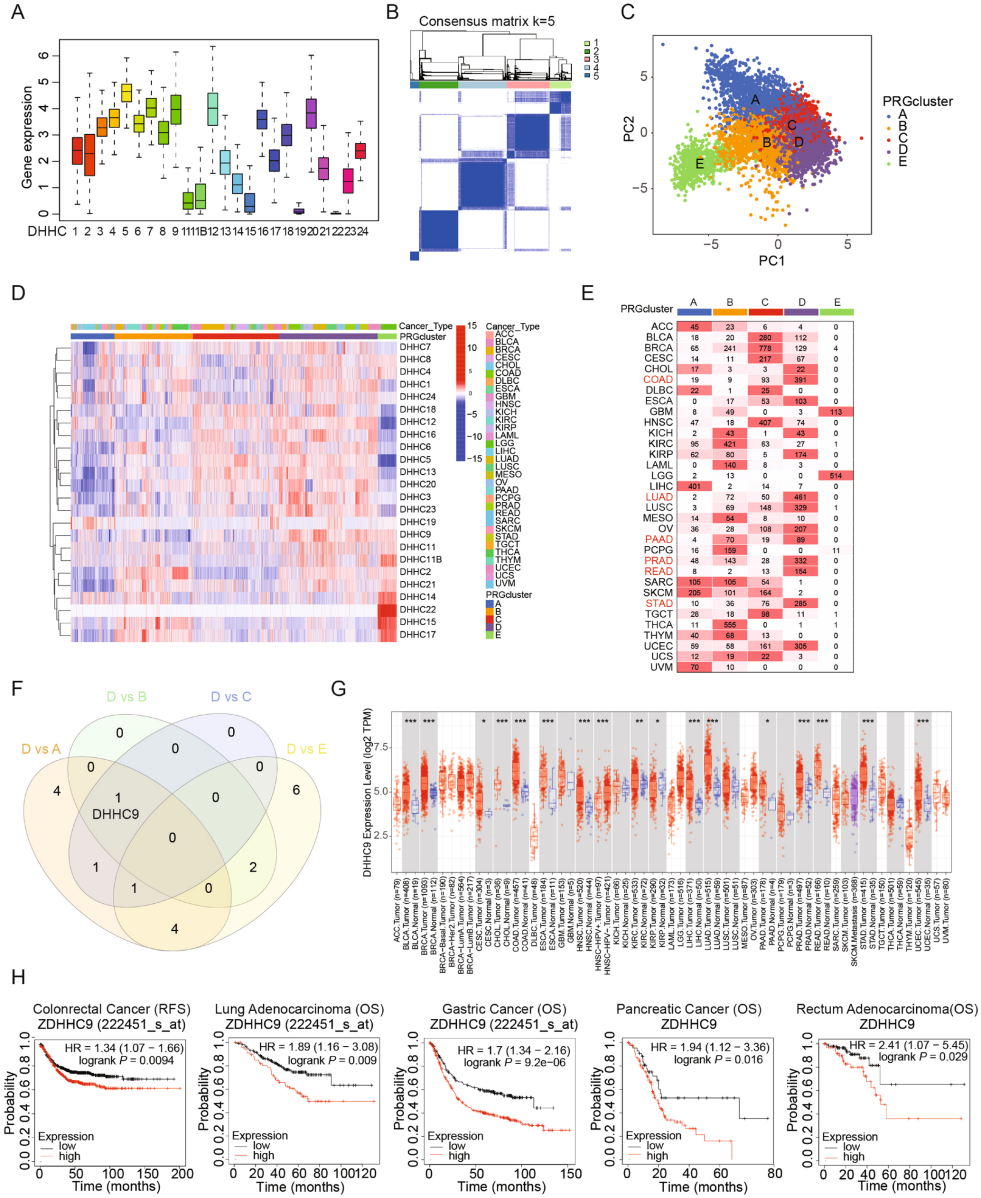
Identification of *DHHC9* as a potential key regulator in adenocarcinoma. (A) Expression patterns of DHHC family members across various cancer types. (B) Unsupervised clustering of DHHC expression profiles across cancer types. Five tumour subgroups (PRGclusters) with distinct DHHC expression patterns were identified. (C) Principal component analysis (PCA) of DHHC expression across 33 cancer types. The clustering pattern from PCA supported the results from unsupervised clustering analysis. (D) Heatmap showing DHHC expression patterns in a pan‐cancer analysis. The heatmap provides a comprehensive view of the varied expression of DHHC enzymes across cancer types. (E) Tumour‐type distribution across PRGclusters. (F) Comparative expression analysis of DHHCs across subgroups. (G) Expression analysis of DHHC9 in tumour types compared to adjacent normal tissues. (H) Clinical significance of DHHC9 expression in various adenocarcinomas.

To investigate the roles of DHHC enzymes in cancer, we analysed the mutation profiles of DHHC family members across 33 cancer types using data from the TCGA cohort, but no significant collective mutations were identified across *DHHC* genes (Figure S1A,B). However, an analysis of copy number variations revealed that several *DHHC* family members, including *DHHC2*, *DHHC4*, *DHHC5*, *DHHC6*, *DHHC9*, *DHHC11*, *DHHC19*, *DHHC21*, *DHHC23* and *DHHC24*, exhibited more frequent amplifications than deletions across various cancers (Figure S1C,D). To better understand the role of DHHC enzymes, we assessed their expression levels across multiple tumour types. This analysis revealed distinct and diverse expression profiles among DHHC family members (Figure S1E). Certain *DHHC*s displayed elevated expression in specific cancer types, suggesting potential cancer‐type‐specific regulatory functions (Figure 1A). Unsupervised clustering analysis further stratified the tumours into five tumour subgroups (PRGclusters) based on *DHHC* expression profiles (Figure 1B). The robustness of this clustering was validated through principal component analysis (PCA) (Figure 1C), and the pan‐cancer expression landscape of the *DHHCs* was visualised through a detailed heatmap (Figure 1D). When the patient samples were categorised into subgroups based on tumour type, subgroup D emerged as being predominantly composed of adenocarcinomas (Figure 1E) and subgroup E exclusively contained brain tumours (GBM and LGG) (Figure 1E). This prompted us to investigate whether specific *DHHC*s were uniquely associated with subgroup D. Comparative expression analysis revealed that *DHHC9* was markedly overexpressed in subgroup D relative to subgroups A, B and C, while its expression was not significantly different from subgroup E, likely due to the unique representation of brain tumours in subgroup E (Figure 1F). To confirm the relevance of *DHHC9* in adenocarcinomas, we analysed its expression in tumour versus adjacent normal tissues. *DHHC9* was consistently overexpressed in tumour tissues across all adenocarcinoma types examined (Figure 1G). Furthermore, elevated *DHHC9* expression was significantly associated with poor clinical outcomes in colorectal cancer, lung adenocarcinoma, gastric cancer, pancreatic cancer and rectal adenocarcinoma (Figure 1H). Taken together, these findings suggest that increased expression of *DHHC9* is closely related to adenocarcinoma development and progression, highlighting it as a potential therapeutic target.

### 3.2 *DHHC9* Promotes Adenocarcinoma Cell Migration Through Palmitoylation

**FIGURE 2:**
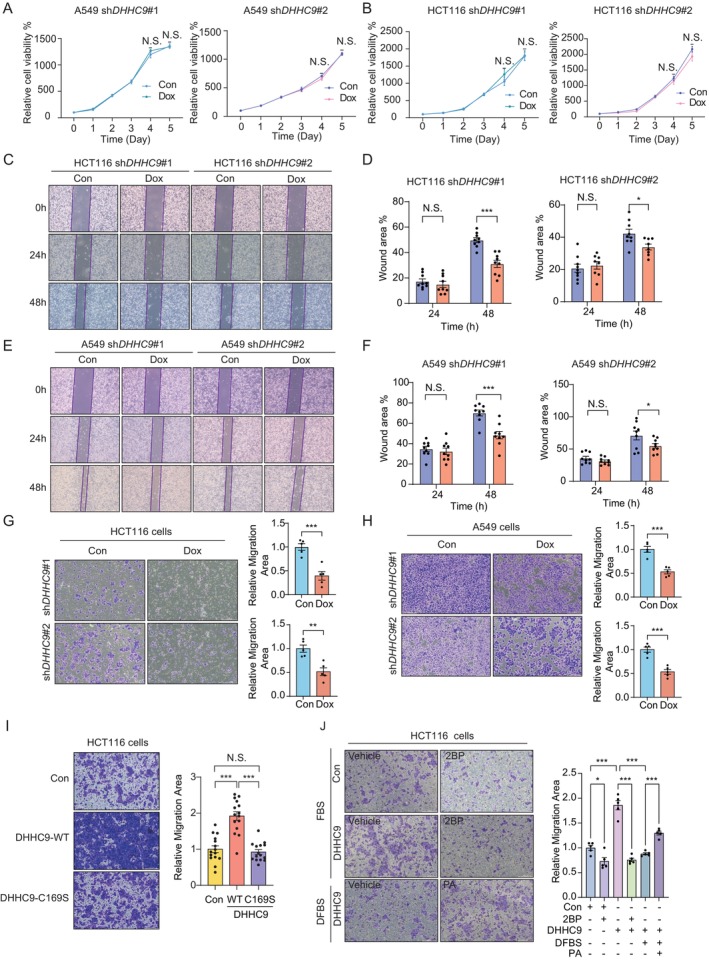
*DHHC9* promotes cell migration through palmitoylation. (A) Cell viability of doxycycline‐induced DHHC9‐knockdown A549 cells measured at indicated times. (Absorbance at 495 nm; mean ± SD, *n* = 6.). (B) Cell viability of doxycycline‐induced DHHC9‐knockdown A549 cells measured at indicated times. (C) Representative results of wound‐healing assays in the adenocarcinomas cell lines HCT116. (D) Quantitative analysis of scratch wound healing assay in (D) after a 48 h treatment of doxycycline. (E) Representative results of wound‐healing assays in the adenocarcinomas cell lines A549. (F) Quantitative analysis of scratch wound healing assay in (E) after a 48 h treatment of doxycycline. (G) Transwell migration assay with the 24‐well Transwell system in HCT116 cells and quantitative analysis of the migration area. (H) Transwell migration assay with the 24‐well Transwell system in A549 cells and quantitative analysis of the migration area. (I) Representative images of transwell migration assays for cells overexpressing Control, DHHC9‐WT, or the catalytically inactive mutant DHHC9‐C169S, along with quantitative analysis of the migrated area. (J) Transwell migration assays of HCT116 cells overexpressing DHHC9 under different conditions: Treatment with 2‐bromopalmitate (2‐BP), culture in dialysed fetal bovine serum (DFBS) and DFBS supplemented with palmitic acid (PA). Data represent the mean ± SEM; statistical significances were determined by unpaired two‐sided Student's *t*‐test. *p < 0.05, **p < 0.01, ***p < 0.001.

To further investigate the role of *DHHC9* in adenocarcinoma, we examined its structural variants, copy number alterations, mutations and mRNA expression levels across various adenocarcinomas using TCGA cohorts. Our analysis revealed that colorectal and lung adenocarcinomas exhibited the highest *DHHC9* expression levels (Figure S2A). To explore the functional role of *DHHC9*, we generated doxycycline‐inducible *DHHC9* knockdown models in human lung cancer A549 cells and colon cancer HCT116 and DLD1 cells, achieving efficient knockdown (Figure S2B). However, cell viability assays revealed that the reduced expression of DHHC9 did not significantly affect the proliferation of these cancer cells (Figure 2A,B; Figure S2C). Given the potential involvement of *DHHC9* in cellular behaviours beyond proliferation, we assessed its role in cell migration. Using wound healing assays, we observed a significant reduction in cell migration following *DHHC9* knockdown in both HCT116 (Figure 2C,D) and A549 cells (Figure 2E,F). The transwell migration assays further confirmed that *DHHC9* knockdown markedly suppressed the migration of invasive cells, whereas doxycycline treatment alone had no observable effect (Figure 2G,H; Figure S2D,E).

To determine whether *DHHC9*‐mediated palmitoylation is critical for its role in migration, we transfected HCT116 cells with either wild‐type (WT) *DHHC9* or a catalytically inactive *DHHC9 C169S* mutant. Remarkably, WT *DHHC9* expression promoted migration, whereas the C169S mutant failed to enhance migration, indicating that the enzymatic activity of *DHHC9* is essential for its role in cell migration (Figure 2I). To further assess the palmitoylation dependence of DHHC9‐driven migration, we performed two complementary assays (Figure 2J). First, pharmacological inhibition with 2‐bromopalmitate (2‐BP), a catalytic site–directed pan‐palmitoyltransferase inhibitor, completely abolished the enhanced migration induced by *DHHC9* overexpression. Second, in a palmitate rescue assay under palmitate‐depleted conditions using dialysed fetal bovine serum (DFBS), DHHC9 overexpression failed to promote migration; supplementation with palmitic acid (PA) restored the pro‐migratory phenotype (Figure 2J).

Together, these results demonstrate that DHHC9 promotes tumour cell migration specifically in a palmitoylation‐dependent manner, underscoring its potential as a therapeutic target for limiting cancer metastasis.

### 3.3 Targeting DHHC9 Suppresses the Tumour Metastasis of Colon Cancer Cells In Vivo

**FIGURE 3:**
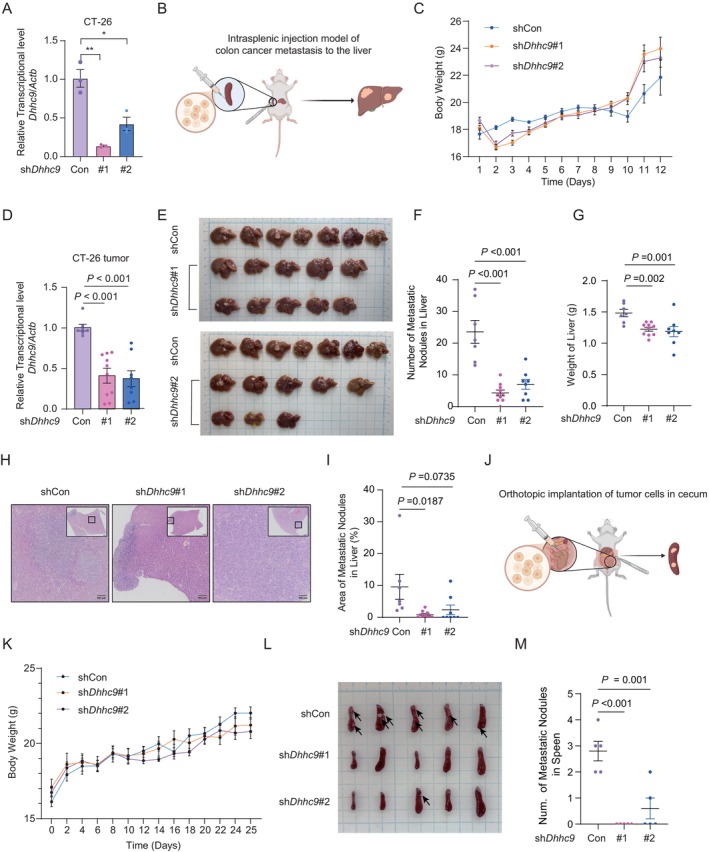
DHHC9 knockdown exerts anti‐tumour effects in vivo. (A) The silencing efficiency of the shDHHC9 vector was validated by qPCR in CT‐26 cells. (B) Schematic diagram for the timeline of the spontaneous spleen transfer to the liver model. (C) Body weights of mice were measured every other day. (D) The DHHC9 level in primary tumours was detected by qPCR. (E) Bright‐field image of the liver was shown. (F) Quantification of liver metastatic nodules from (E). (G) The weight of the liver in each group was measured. (H) Representative H&E staining of the indicated metastatic tumour in the liver was shown. (I) Percentage of the metastatic tumour area in (g). (J) Schematic diagram for the timeline of the orthotopic co‐implantation colon tumour model. (K) Body weights of mice in the orthotopic co‐implantation colon tumour model were measured every other day. (L) Image of the spleens with mice inoculated with CT‐26 cells in the cecum was shown. (M) The number of surface metastatic nodules in the spleen was calculated. For (C) and (K), statistical significance was determined by two‐way ANOVA. For (A, D, F, G, I and M), data represent the mean ± SEM; statistical significance was determined by an unpaired two‐sided Student's *t*‐test.

Building on our in vitro findings that *DHHC9* silencing inhibits adenocarcinoma cell migration, we sought to evaluate whether DHHC9 knockdown could effectively suppress tumour metastasis in vivo. To investigate this, we established CT‐26 mouse colon cancer cells stably expressing either *DHHC9*‐specific shRNA (sh*DHHC9*) or a control shRNA (shCon) vector (Figure 3A). The in vivo effects of *DHHC9* knockdown were assessed using a well‐established spontaneous spleen‐to‐liver metastasis model (Figure 3B). In this model, genetically modified CT‐26 cancer cells injected into the spleen disseminate to the liver, mimicking the natural metastatic progression of colon cancer. To ensure that *DHHC9* knockdown did not negatively impact overall health, body weight was monitored throughout the experiment, and no significant differences were observed between the shDHHC9 and control groups, indicating that *DHHC9* silencing does not impair systemic metabolic homeostasis (Figure 3C). Two weeks after tumour cell inoculation, the mice were sacrificed, and spleen and liver tissues were collected for further analysis. Quantitative PCR (qPCR) confirmed efficient *DHHC9* knockdown in primary tumours within the spleen (Figure 3D), validating the successful establishment of the knockdown model. Consistent with our in vitro findings, *DHHC9* silencing led to a significant reduction in liver metastases compared to the control group (Figure 3E,F). To further quantify the metastatic burden, liver weights were measured as an indicator of tumour load, showing a marked decrease in the sh*DHHC9* group compared to control (Figure 3G). Histopathological analysis of liver tissue sections corroborated these findings, revealing significantly fewer metastatic foci in the livers of sh*DHHC9* mice compared to controls, further supporting the suppressive effect of *DHHC9* knockdown on metastatic progression (Figure 3H,I).

In addition to the spontaneous spleen‐to‐liver metastatic model, we also established an orthotopic implantation model, in which tumour cells were implanted into the cecum (Figure 3J). Body weights monitored throughout the experiment revealed no significant differences between the sh*DHHC9* and control group, further confirming that *DHHC9* silencing does not adversely affect systemic health (Figure 3K). Three weeks post‐implantation, tumours metasized to the spleen in the control group (Figure 3L). Notably, *DHHC9* knockdown resulted in a significant reduction in spleen metastases compared to controls (Figure 3M). Furthermore, a significant increase in ascitic fluid accumulation was observed in the control group compared to the shDHHC9 group, suggesting that *DHHC9* knockdown effectively blocks tumour cell dissemination within the abdominal cavity. In summary, these findings provide compelling evidence that DHHC9 plays a critical role in promoting colon cancer metastasis. Its targeted inhibition represents a promising therapeutic strategy for preventing disease progression.

### 3.4 Refined ABE Approach Facilitates DHHC9‐Specific Chemical Proteomic Substrate Mapping

**FIGURE 4:**
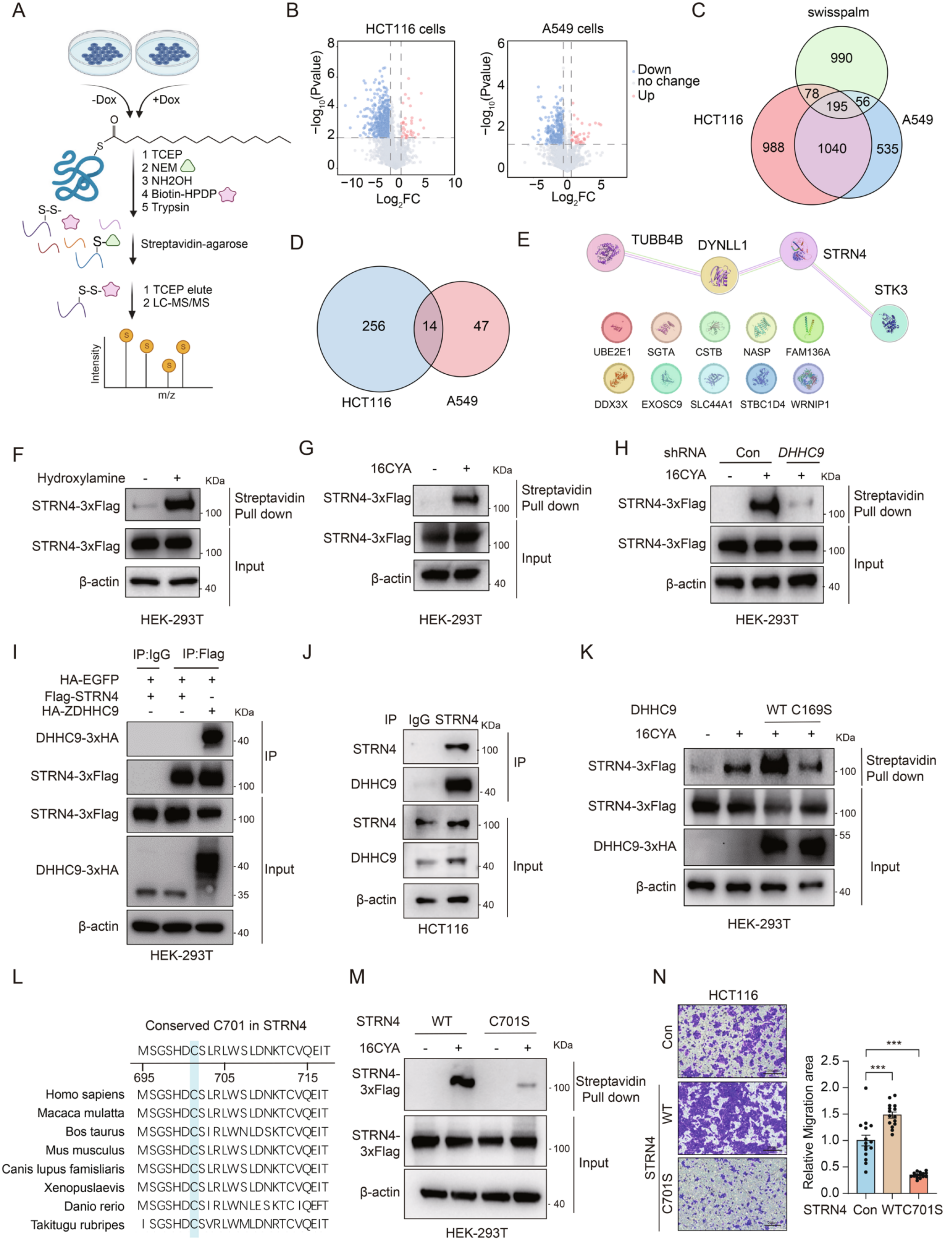
STRN4 as a key substrate of DHHC9 in promoting adenocarcinoma cell migration. (A) Illustration of the Modified ABE workflow for large‐scale S‐acylated protein identification. (B) Volcano plot of the S‐palmitoylsome identifying the substrate of DHHC9 in HCT116 and A549 cells. Fold changes (FC) of proteins' abundance in DHHC9 knockdown versus control samples are shown as a function of significance. Significance cutoffs were *p* = 0.01 (unpaired two‐tailed Student's *t*‐test) and FC = 2 (HCT116 cells) or 1.5 (A549 cells). *n* = 3. (C) *S*‐palmitoylated proteins identified by modified ABE in HCT116 and A549 cells were compared with known *S*‐palmitoylated proteins with modification site information in the SwissPalm database. (D) Venn diagram of identified DHHC9 substrate maps in A549 and HCT116 cells. (E) STRING protein–protein interaction network of DHHC9 substrates shared by HCT116 and A549 cells. (F) STRN4 palmitoylation was validated in lysates derived from Flag‐tagged STRN4 overexpressed in HEK293T cells using the ABE method. (G) HEK293T cells were metabolically labelled with 50 μM alkynyl PA (16CYA) overnight. Flag‐STRN4 palmitoylation levels were analysed by click reaction and streptavidin bead pulldown, followed by immunoblotting. (H) Western blot analysis of STRN4 palmitoylation in HEK293T cells after knockdown of endogenous *DHHC9* using shRNA plasmids. (I) Co‐IP assays of overexpressed Flag‐STRN4 and HA‐DHHC9 in HEK‐293T cells, showing the interaction. (J) Co‐IP assay of endogenous DHHC9 and STRN4 in HCT116 cells. (K) Palmitoylation analysis showing that catalytically inactive DHHC9 C169S did not induce STRN4 palmitoylation. (L) Alignment of the STRN4 protein sequence among different species. (M) HEK293T cells were transfected with Flag‐tagged wild‐type STRN4 or the STRN4 (C701S) mutant and metabolically labelled with 16CYA. Palmitoylation was analysed by western blot. (N) Transwell assay and the quantification result showing the changes in migration rate of wild‐type STRN4 and STRN4 (C701S) mutant in HEK293T cell lines.

Given the distinct substrate specificities of DHHC enzymes across tissues and cell types, identifying DHHC9‐specific substrates remains a significant challenge. To address this, we developed a novel, integrated mass spectrometry‐based strategy to identify substrates and S‐acylation sites specifically modified by DHHC9. Using control and doxycycline‐induced DHHC9 knockdown HCT116 and A549 cells, we applied a multiplex approach combining a modified acyl‐biotin exchange (ABE) technique with label‐free quantification for comprehensive S‐palmitoylation analysis (Figure 4A). In this optimised workflow, *N*‐ethylmaleimide (NEM) and 2,2′‐dithiodipyridine (DTDP) were used to block free and disulfide‐bonded cysteines, ensuring accurate substrate identification. Hydroxylamine selectively cleaved S‐palmitoyl groups, followed by biotin‐HPDP labeling and trypsin digestion. The biotinylated peptides, representing previously S‐acylated proteins, were enriched via streptavidin affinity purification and eluted with TCEP. This innovative approach minimises nonspecific binding while precisely identifying DHHC9‐specific S‐acylation sites through reduced cysteine residues. Our methodology provides a robust and reproducible pipeline for profiling DHHC9‐mediated S‐palmitoylation, offering new insights into its role in cancer biology.

Using this approach, we identified 8168 unique S‐palmitoylated peptides corresponding to 2301 proteins across three biological replicates of control and *DHHC9* knockdown HCT116 cells. Among these, palmitoylation of 270 proteins was significantly downregulated in *DHHC9* knockdown HCT116 cells compared to control cells, indicating that these proteins are potential substrates of DHHC9 palmitoyl transferase (Figure 4B, Table S1). Extending this analysis to A549 cell lines, we identified 5069 S‐palmitoylated peptides from 1826 proteins, including 61 potential DHHC9 substrates (Figure 4B, Table S2). Notably, 2563 proteins (2028 in HCT116 and 1575 in A549) were newly identified S‐palmitoylated proteins with palmitoylated site information compared to the SwissPalm database (1319 proteins with palmitoylated sites information) (Figure 4C). We also conducted Gene Ontology (GO) enrichment analysis for cellular compartments and biological processes for DHHC9 substrates to further examine the localisation and functional diversity among DHHC9 substrates in HCT116 and A549 cells (Figure S3A–D). We found that substrates were significantly over‐represented (FDR < 0.05) in compartments like focal adhesion, cell–substrate junction, cytoplasmic vesicle and secretory granule, aligning with known DHHC9 cellular localisation (Figure S3A,C). Analysis of biological processes showed enrichment in regulation of mRNA or amino acid metabolic process, translation and viral process in HCT116 cells and A549 cells (Figure S3B,D), highlighting a distinct profile compared to the general proteome. To identify the key DHHC9 substrates implicated in adenocarcinoma metastasis, we further analysed the common 14 substrates in both HCT116 and A549 cell lines (Figure 4D). To delineate the substrate network regulated by DHHC9, we conducted a STRING protein–protein interaction analysis. This analysis revealed a functionally clustered network comprising STRN4, STK3, DYNLL1 and TUBB4B (Figure 4E), all of which are established regulators of cell migration.

### 3.5 STRN4 as Key Substrate of DHHC9 Driving Adenocarcinoma Cell Migration

Among the four candidate substrates identified, STRN4, a core regulatory B subunit of protein phosphatase 2A (PP2A) within the striatin‐interacting phosphatase and kinase (STRIPAK) complex, emerged as a priority target. The STRIPAK complex, via its adaptor STRN4, is known to suppress Hippo pathway kinases STK3/STK4 (MST1/2) and MAP4Ks, implicating it in tumorigenesis [32]. We hypothesised that DHHC9‐mediated palmitoylation of STRN4 might regulate its migratory function in adenocarcinoma. Using acyl‐biotin exchange (ABE) assays and metabolic labeling with alkyne‐conjugated palmitic acid (16CYA), we confirmed STRN4 as an S‐palmitoylated protein (Figure 4F,G). To establish DHHC9's role in this modification, we employed doxycycline‐inducible and transient shRNA‐mediated DHHC9 knockdown in HEK293T cells, both of which significantly reduced STRN4 palmitoylation (Figure 4H, Figure S3E–G). Co‐immunoprecipitation assays further demonstrated a direct physical interaction between DHHC9 and STRN4 using both overexpressed (Figure 4I) and endogenous proteins (Figure 4J). Overexpression of wild‐type DHHC9, but not the catalytically inactive C169S mutant, enhanced STRN4 palmitoylation (Figure 4K), confirming DHHC9 as the responsible palmitoyltransferase. Cross‐species sequence alignment identified cysteine 701 (C701) as a conserved palmitoylation site (Figure 4L). Substituting C701 with serine (C701S) abolished STRN4 palmitoylation (Figure 4M), validating this residue as critical for modification. Functional assays in HCT116 cells revealed that wild‐type STRN4 robustly enhanced cell migration, whereas the C701S mutant lacked this activity (Figure 4N). These findings demonstrate that DHHC9‐mediated palmitoylation of STRN4 at C701 is essential for its ability to promote adenocarcinoma cell migration. This post‐translational modification dynamically regulates STRN4's function within the STRIPAK complex, positioning it as a pivotal mediator of DHHC9‐driven metastasis.

### 3.6 STRN4 Palmitoylation Directs Phosphatase Activity and Promotes Cell Migration via the STRN4‐YAP Pathway in Adenocarcinoma Cells

**FIGURE 5:**
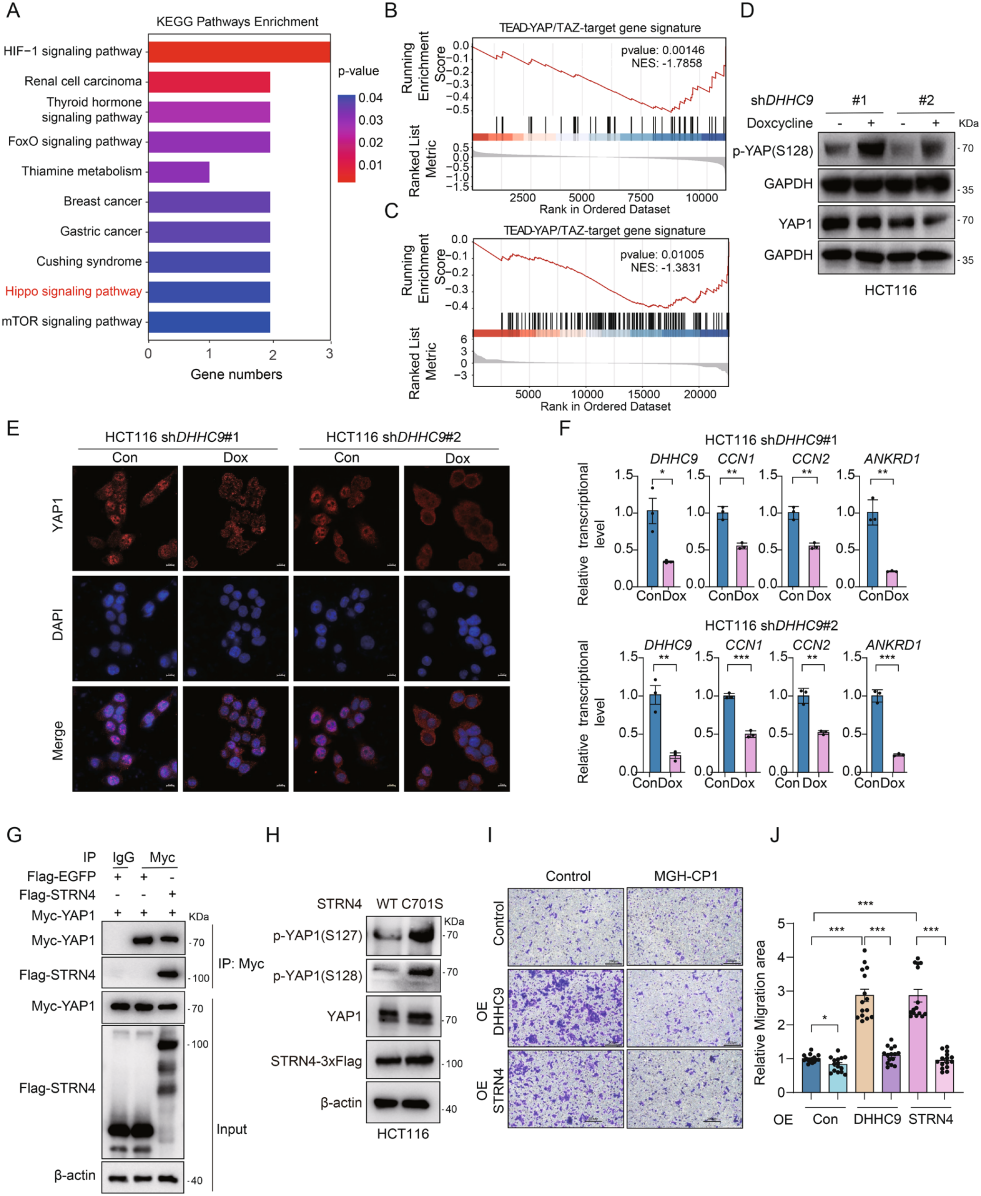
DHHC9 promotes cell migration through the Hippo–YAP signalling pathway. (A) KEGG pathway enrichment analysis of the differentially expressed genes of doxycycline‐induced *DHHC9* knockdown HCT116 cells compared to control cells. (B) Gene set enrichment analysis of YAP/TAZ‐TEAD target gene signature after *DHHC9* knockdown. (C) GSEA enrichment plot of TEAD‐YAP/TAZ target genes in HCT116 cells overexpressing wild‐type STRN4 (STRN4‐WT) versus the palmitoylation‐deficient mutant STRN4‐C701S. (D) Effect of *DHHC9* knockdown on expression and phosphorylation of YAP in HCT116 cells. (E) YAP localisation was detected with confocal laser scanning microscopy in doxycycline‐induced DHHC9 knockdown HCT116 cells. (F) Knockdown of DHHC9 in HCT116 cells suppressed YAP downstream gene transcription. (G) Co‐immunoprecipitation assays of overexpressed Flag‐STRN4 and Myc‐YAP1 in HEK293T cells, with EGFP‐Flag used as a control. (H) Western blot analysis of the effect of STRN4‐WT and palmitoylation‐deficient STRN4‐C701S overexpression on YAP phosphorylation in HCT116 cells. (I) Transwell assay showing changes in migration rate after MGH‐CP1 treatment in HCT116 cells transfected with DHHC9 and STRN4 plasmids. Scale bars: 100 μm, *n* = 3 biologically independent experiments. (J) Statistical data presented in results (I). For (A), Pathways with a *p* value < 0.05 (by two‐sided hypergeometric test) and the inclusion of at least two genes were summarised. For (F) and (I), data represent the mean ± SEM (*n* = 3 biologically independent samples); statistical significances were determined by unpaired two‐sided Student's *t*‐test.

Metastasis remains the primary cause of cancer‐related mortality, with effective therapeutic strategies still limited. The transcriptional co‐activators YAP and TAZ have been identified as central mediators of metastatic progression, driving critical processes such as epithelial‐to‐mesenchymal transition (EMT), tumour cell invasion and adaptation to the metastatic microenvironment [33, 34, 35]. Recent studies further implicate STRN4 as a key positive regulator of the Hippo pathway, facilitating YAP activation [32]. Despite the recognised importance of protein phosphatases in oncogenic signalling, their regulatory mechanisms remain less explored compared to kinases [36]. Specifically, the pathways governing STRN4's role in metastasis are poorly understood. We hypothesised that DHHC9‐mediated palmitoylation of STRN4 enhances its phosphatase activity, thereby amplifying YAP/TAZ‐driven signalling to promote metastatic behaviour. To investigate this, we first tested the effect of STRN4 palmitoylation on Hippo‐YAP signalling on doxycycline‐induced *DHHC9* knockdown HCT116 cells. KEGG pathway enrichment analysis of RNA sequencing (RNA‐seq) revealed several enriched signalling pathways, with the Hippo pathway being prominently affected after DHHC9 knockdown (Figure 5A). Further Gene Set Enrichment Analysis (GSEA) using a predefined TEAD‐YAP/TAZ target gene signature demonstrated that *DHHC9* knockdown significantly suppressed TEAD‐YAP/TAZ target genes, with a normalised enrichment score of −1.7858 (Figure 5B). Consistent with these findings, RNA‐seq analysis of HCT116 cells overexpressing STRN4‐WT versus the palmitoylation‐deficient mutant STRN4‐C701S revealed significant enrichment of TEAD–YAP/TAZ signalling signatures (NES = −1.3831; Figure 5C). Furthermore, Fisher's exact test of transcriptome‐wide data identified a significant overlap between genes altered by DHHC9 knockdown and those differentially expressed in STRN4‐C701S– versus STRN4‐WT–expressing cells (Figure S4A). Notably, correlation analysis of YAP signalling–related genes affected in both contexts (DHHC9 knockdown vs. DHHC9 control and STRN4‐C701S vs. STRN4‐WT) showed a significant positive correlation (*p* = 0.0433; Figure S4B), reinforcing that DHHC9‐mediated palmitoylation of STRN4 directly modulates YAP activity in HCT116 cells. Western blot analysis further confirmed these findings by showing increased phosphorylation of YAP1 in *DHHC9*‐knockdown HCT116 cells, indicating reduced YAP activity (Figure 5D). Additionally, immunofluorescence (IF) staining with YAP‐specific antibodies revealed that *DHHC9*‐knockdown impaired YAP nuclear localisation (Figure 5E). In line with these observations, the expression levels of key YAP/TAZ target genes, including *CCN1* (Cyr61), *CCN2* (CTGF) and *ANKRD1*, were downregulated in a *DHHC9*‐dependent manner, further supporting the notion of reduced YAP activity (Figure 5F, Figure S4C,D). To investigate how palmitoylated STRN4 affects YAP signalling, we examined the interaction between STRN4 and YAP. Co‐immunoprecipitation assays revealed that STRN4 directly binds YAP (Figure 5G). Moreover, overexpression of wild‐type (WT) STRN4, but not the palmitoylation‐deficient C701S mutant, enhanced YAP dephosphorylation and activity in HCT116 cells (Figure 5H, Figure S4E). This mirrors DHHC9 knockdown phenotypes, confirming that palmitoylation governs STRN4's ability to scaffold PP2A and suppress Hippo kinases. Finally, to determine the functional consequences of this regulation on cell migration, we employed the TEAD inhibitor MGH‐CP1, which blocks YAP activation by preventing YAP/TAZ‐TEAD interactions. As expected, the enhanced migratory effects driven by DHHC9 and STRN4 overexpression were effectively reversed by MGH‐CP1 treatment (Figure 5I,J, Figure S4F). Notably, MGH‐CP1 treatment did not affect cell viability, confirming that its effects were specific to migration inhibition (Figure S4G). In summary, our findings establish that DHHC9‐mediated palmitoylation of STRN4 as a regulatory switch controlling STRN4's phosphatase function, thereby activating YAP/TAZ‐driven metastasis. These underscore intervening DHHC9 or STRN4 palmitoylation as promising therapeutic strategies for combating Hippo‐YAP‐driven adenocarcinoma metastasis.

### 3.7 Identification of Treprostinil and 10‐Hydroxycamptothecin as Potential DHHC9 Inhibitors for Suppressing Adenocarcinoma Cell Migration

**FIGURE 6:**
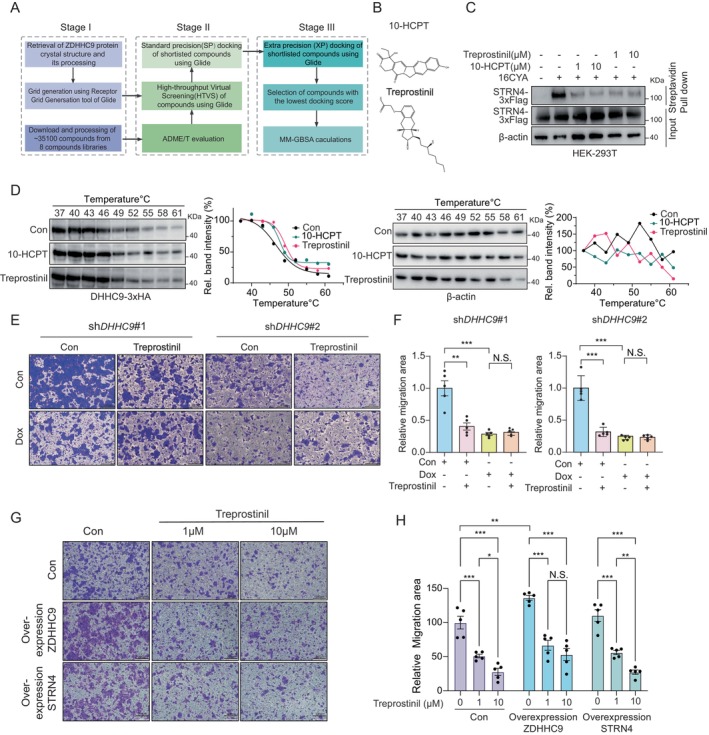
Identifying potential DHHC9 inhibitors through virtual screening for anti‐metastatic drug discovery. (A) Overall workflow for structure‐based virtual screening to identify novel DHHC9 inhibitors. (B) Chemical structures of the two potential DHHC9 inhibitors, 10‐HPCT and treprostinil. (C) Effect of 10‐Hydroxycamptothecin and Treprostinil on STRN4 palmitoylation. (D) Immunoblotting‐based thermal shift assay suggesting treprostinil (20 μM) and 10‐HCPT (10 μM) stabilised DHHC9 in HCT116 cell lysates. (E) Transwell assay for the detection of cell migration after treprostinil treatment in doxycycline‐induced DHHC9‐knockdown HCT116 cells. (F) Statistical data presented in results (f). (G) Transwell assay of Treprostinil‐treated HCT116 cells after Flag‐DHHC9 or Flag‐STRN4 overexpression. (H) Quantitative analysis of the migration area in (G). For (F) and (H), data represent the mean ± SEM, *n* = 5, statistical significances were determined by unpaired two‐sided Student's *t*‐test.

Given the crucial role of DHHC9 in promoting tumour metastasis, we explored the feasibility of inhibiting its function through small‐molecule targeted intervention. Increasing evidence suggests that targeting protein palmitoylation through DHHC inhibition holds therapeutic potential for cancer and other diseases, positioning DHHC enzymes as promising drug targets [21, 37]. However, despite being identified two decades ago and recent advances in structural elucidation, clinically viable DHHC inhibitors remain scarce. Among the currently available DHHC inhibitors, 2BP is the most widely used [38]; however, its low potency, high cytotoxicity and poor selectivity limit its therapeutic applications. The lack of potent and selective DHHC inhibitors presents a significant challenge in advancing DHHC‐related research and therapeutic development. To address this, we performed virtual screening of the Bioactive Molecules Database from Tsinghua University's Bioactive Compounds and Repurposing Libraries, containing 35,143 compounds, to identify novel inhibitors targeting DHHC9 (PDB ID: 8HF3). Following an initial screening based on Lipinski's rule of five and ADME/T (absorption, distribution, metabolism, excretion and toxicity) properties, 19,486 compounds were selected for further analysis. The selected compounds were prepared for docking using the LIGPREP tool and underwent a hierarchical docking process, including high‐throughput virtual screening (HTVS), standard precision (SP) and extra precision (XP) docking, followed by MM‐GBSA free energy calculations to refine the potential hits (Figure 6A). Ultimately, the top 30 compounds with the highest XP GScore were subjected to molecular biology and functional validation assays (Figure S5A). Among these, molecular docking studies revealed that 10‐Hydroxycamptothecin (10‐HCPT) and Treprostinil specifically interacted with DHHC9. Particularly, Treprostinil binds to amino acid residues 166–169, exhibiting the lowest binding free energy of −60.75 kcal/mol (Figure S5B). Functional validation validated that both 10‐Hydroxycamptothecin (10‐HCPT) and Treprostinil emerged as potent inhibitors that effectively suppressed the palmitoylation of its substrate STRN4, whereas three other tested compounds showed no significant effects (Figure 6B, Figure S5C).

Further validation experiments confirmed that both 10‐HCPT and Treprostinil significantly inhibited DHHC9 and STRN4 palmitoylation at concentrations of 1 and 10 μM (Figure 6C, Figure S5D). The binding of Treprostinil and 10‐HCPT to DHHC9 was further validated through a thermal shift assay, which demonstrated that both Treprostinil and 10‐HCPT stabilised DHHC9 across a range of temperatures using cell lysates (Figure 6D). Considering that Treprostinil is already clinically approved for the treatment of primary pulmonary hypertension, we investigated its potential for selectively inhibiting DHHC9 and its downstream effects on cancer cell migration. Transwell migration assays demonstrated that Treprostinil significantly reduced HCT116 cell migration. However, DHHC9 silencing abolished this inhibitory effect, indicating that Treprostinil specifically targets DHHC9‐dependent pathways (Figure 6E,F). Consistently, overexpression experiments in HCT116 cells revealed that Treprostinil treatment effectively reversed the migratory effects induced by DHHC9 and STRN4 overexpression, without prominently affecting cell viability (Figure 6G,H, Figure S5E). Comparable results were observed with 10‐HCPT treatment, except for cell cytotoxicity at the 48‐h time point, further supporting its role as a DHHC9 inhibitor (Figure S5F–H). In conclusion, we identified Treprostinil and 10‐HCPT as promising DHHC9 inhibitors, capable of suppressing adenocarcinoma cell migration by blocking the palmitoylation of DHHC9 and its substrate STRN4. These results highlight the therapeutic potential of DHHC9 inhibition in combating cancer metastasis.

### 3.8 In Vivo Efficacy of Treprostinil and 10‐HCPT in Suppressing Colon Cancer Metastasis

**FIGURE 7:**
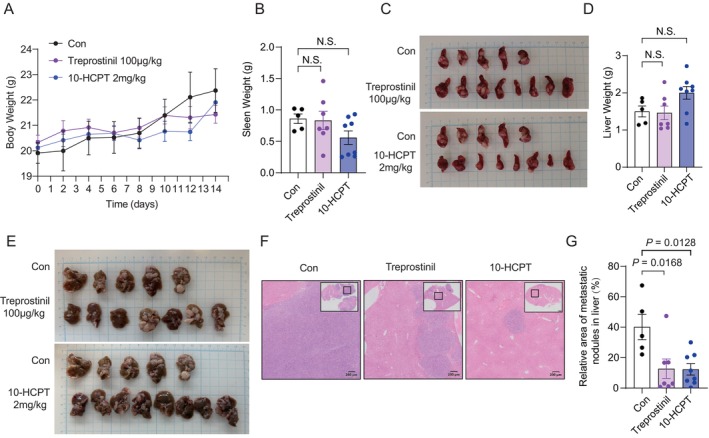
Pharmacological inhibition of DHHC9 by Treprostinil or 10‐HCPT suppresses colon cancer metastasis in vivo. (A) Body weights of vehicle‐, Treprostinil (100 μg/kg)‐ and 10‐HCPT (2 mg/kg)‐treated mice measured every other day. (B) Spleen weights for each group. (C) Representative bright‐field images of spleens. (D) Liver weights for each group. (E) Representative bright‐field images of livers. (F) Representative H&E staining of metastatic tumours in liver sections. (G) Quantification of metastatic tumour area shown in (F). Data in (B), (D) and (G) represent mean ± SEM; statistical significance was determined by unpaired two‐sided Student's *t*‐test.

To evaluate the therapeutic potential of the identified DHHC9 inhibitors in vivo, we treated mice bearing CT‐26 colon cancer cells in the spontaneous spleen‐to‐liver metastasis model with Treprostinil (100 μg/kg) or 10‐HCPT (2 mg/kg) via intraperitoneal injection, alongside a vehicle control group (Figure 7A–G). Throughout the 14‐day experimental period, no significant differences in body weight were observed between the treatment groups and vehicle controls (Figure 7A), indicating minimal systemic toxicity of both compounds at the administered doses.

Analysis of primary tumour burden revealed no significant difference in spleen weight between the inhibitor‐treated groups and vehicle controls (Figure 7B,C), consistent with our earlier findings that DHHC9 knockdown does not affect primary tumour growth. Similarly, while liver weights remained unchanged across all groups (Figure 7D). However, histopathological analysis revealed marked suppression of metastatic burden in treated mice. Bright‐field imaging showed visibly reduced metastatic nodules on liver surfaces following Treprostinil or 10‐HCPT treatment (Figure 7E), and H&E‐stained sections confirmed a substantial decrease in metastatic tumour area (Figure 7F,G). Collectively, these findings demonstrate that pharmacological inhibition of DHHC9 by either Treprostinil or 10‐HCPT effectively suppresses colon cancer metastasis in vivo without compromising systemic health or primary tumour growth, providing compelling preclinical support for targeting the DHHC9‐STRN4‐YAP axis to limit adenocarcinoma dissemination.

## 4 Discussion

In this study, we identified DHHC9 as a key driver of adenocarcinoma metastasis and demonstrated its essential role in promoting cancer cell migration through STRN4 palmitoylation. By integrating proteomic analysis with functional assays, we provide compelling evidence that DHHC9‐mediated STRN4 palmitoylation facilitates YAP nuclear localisation, thereby enhancing YAP/TAZ signalling and ultimately driving cancer cell migration. Furthermore, through a comprehensive small‐molecule virtual screening approach, we identified two potent DHHC9 inhibitors, Treprostinil and 10‐HCPT, which effectively suppress DHHC9 activity and inhibit adenocarcinoma cell migration (Figure S6). These findings establish DHHC9 as a promising therapeutic target and lay a strong foundation for further exploration of palmitoylation‐based cancer therapies.

Our findings build upon previous research that highlights the critical role of protein palmitoylation in cancer progression, with several DHHC enzymes identified as oncogenic drivers [7, 11, 39]. Targeting DHHC proteins has emerged as a promising therapeutic strategy for cancer treatment [14, 16, 40]. Among these, DHHC9 has been recognised as an oncogenic driver in various cancers, including brain tumours and leukaemia [41, 42]. Recent studies have further demonstrated that targeting DHHC9 can enhance immune checkpoint blockade (ICB) immunotherapy by regulating PD‐L1 palmitoylation and indirectly modulating the tumour microenvironment [43, 44]. Additionally, DHHC9 has been implicated in the palmitoylation of TIM3, which promotes immune exhaustion and suppresses anti‐tumour immunity [45]. DHHC9‐mediated GLUT1 S‐palmitoylation also promotes glioblastoma glycolysis and tumorigenesis [41]. This suggests that DHHC9 influences multiple tumourigenic pathways beyond metabolism, providing a broader therapeutic rationale for its inhibition. Despite these advances, the specific role of DHHC9 in adenocarcinoma metastasis has remained poorly understood. Our study provides significant insights by elucidating a novel molecular mechanism through which DHHC9 drives tumour progression via STRN4 palmitoylation, leading to phosphatase‐mediated YAP dephosphorylation and activation. Critically, this defines the DHHC9‐STRN4‐YAP axis, establishing a direct mechanistic link between palmitoylation and metastatic potential in adenocarcinoma, while reinforcing STRN4's scaffolding role within the STRIPAK complex.

Recent studies have revealed that multiple ZDHHC family members can modulate Hippo/YAP signalling through palmitoylation of distinct upstream regulators. For example, ZDHHC7‐mediated palmitoylation of the polarity protein Scribble (SCRIB) is essential for its membrane localisation and tumour‐suppressive function; loss of this modification mislocalises SCRIB, leading to Hippo pathway inhibition and YAP activation [46]. Similarly, ZDHHC15 has been shown to palmitoylate KIBRA, an upstream Hippo activator, thereby promoting its degradation and contributing to a palmitic acid–driven ZDHHC15–YAP positive feedback loop that enhances metastatic potential [47]. In contrast to these kinase‐regulatory mechanisms, our study identifies DHHC9‐mediated palmitoylation of STRN4—a core scaffold of the STRIPAK phosphatase complex—as a novel phosphatase‐centred mechanism for YAP activation. Palmitoylation of STRN4 at C701 enhances its ability to recruit PP2A to YAP directly, thereby promoting YAP dephosphorylation and transcriptional activation of metastasis‐associated genes. These findings expand the current understanding of how distinct ZDHHC enzymes converge on the Hippo/YAP pathway via different molecular entry points, highlighting DHHC9–STRN4 as a complementary and previously unrecognised regulatory axis with therapeutic potential in adenocarcinoma metastasis.

In addition to STRN4, our proteomic analysis identified 14 DHHC9‐dependent palmitoylated proteins common to both HCT116 and A549 cells (Figure 4E). Among these, STK3, DYNLL1 and TUBB4B emerge as strong mechanistic candidates for mediating DHHC9‐driven migration and metastasis. STK3 (MST2) is the core Hippo kinase, and its palmitoylation could attenuate Hippo pathway activity in parallel with the STRN4–PP2A axis described here. DYNLL1 and TUBB4B are components of cytoskeletal and trafficking machinery, where palmitoylation could influence focal adhesion turnover, vesicular transport and protrusion dynamics, thereby complementing the transcriptional reprogramming induced by YAP activation. The remaining proteins (e.g., UBE2E1, SGTA, CSTB, NASP, DDX3X, EXOSC9, SLC44A1, STBC1D4, WRNIP1) are less directly linked to metastasis but participate in processes such as ubiquitination, protein quality control, RNA metabolism and DNA repair. Although their precise contributions to adenocarcinoma progression remain to be determined, their palmitoylation by DHHC9 suggests the existence of a broader substrate network that may enhance tumour cell fitness, adaptability and metastatic potential. While STRN4 causality is firmly established in this study, elucidating the functional significance of these additional substrates will require targeted validation in future work. Given DHHC9's ability to modify multiple effectors across distinct oncogenic pathways, direct targeting of DHHC9 may offer a more comprehensive therapeutic strategy than approaches aimed at individual downstream proteins. Furthermore, our identification of potent pharmacological inhibitors presents a promising translational opportunity for the development of palmitoylation‐targeted therapies aimed at curbing cancer metastasis. The identification of two potent DHHC9 inhibitors, Treprostinil and 10‐Hydroxycamptothecin (10‐HCPT), occurred through virtual screening and biochemical validation. Treprostinil, an FDA‐approved drug, offers significant translational potential for rapid repurposing in cancer therapy. Our findings are further supported by robust in vitro and in vivo models, demonstrating that DHHC9 inhibition effectively suppresses metastasis without affecting systemic health. Additionally, the potential synergy of DHHC9 inhibitors with existing therapies, such as immune checkpoint blockade, underscores their clinical relevance. These findings establish DHHC9 as a promising therapeutic target and pave the way for future palmitoylation‐based cancer treatments.

Despite the promising results achieved in this study, several limitations must be acknowledged.

While our in vitro and in vivo models provide valuable mechanistic insights, they may not fully recapitulate the complexity of the tumour microenvironment in human adenocarcinomas. Factors such as immune cell interactions, stromal components and metabolic heterogeneity could influence the therapeutic efficacy of DHHC9 inhibition. Additionally, although Treprostinil and 10‐HCPT demonstrated promising anti‐migratory effects, the pharmacokinetics and off‐target effects of Treprostinil and 10‐HCPT (e.g., 10‐HCPT's topoisomerase inhibition) require further evaluation. Mechanistically, the structural consequences of STRN4 palmitoylation within the STRIPAK complex, such as conformational changes or interaction dynamics, remain unresolved. Finally, DHHC9's broader oncogenic roles, including immune modulation via PD‐L1 or other substrates (e.g., STK3, DYNLL1), were not explored, leaving gaps in understanding its multifaceted contributions to cancer progression.

Future research should aim to address these challenges by employing patient‐derived organoid models and xenograft systems that better recapitulate the clinical tumour microenvironment. Additionally, structure‐guided drug optimisation with improved pharmacological properties will mitigate off‐target effects, and system‐level analyses of DHHC9 substrate networks may reveal new therapeutic vulnerabilities. Based on our findings, we propose that targeting DHHC9‐mediated palmitoylation represents a promising therapeutic strategy for limiting adenocarcinoma metastasis. Given the growing recognition of palmitoylation as a critical regulator of oncogenic signalling, further exploration of other DHHC enzymes and their substrates may uncover additional actionable targets. Moreover, combining DHHC9 inhibitors with existing targeted therapies or immunotherapies could provide synergistic effects, potentially overcoming resistance mechanisms and improving clinical outcomes in metastatic cancer.

In conclusion, our study provides critical insights into the role of DHHC9 in adenocarcinoma metastasis and highlights its potential as a therapeutic target through the inhibition of STRN4 palmitoylation and YAP activation. The identification of Treprostinil and 10‐HCPT as potent DHHC9 inhibitors opens new avenues for the development of palmitoylation‐targeted cancer therapies, while mechanistic insights into lipid‐dependent phosphatase regulation open avenues for targeting ‘undruggable’ pathways. By addressing the outlined limitations and pursuing interdisciplinary strategies, this work lays the foundation for palmitoylation‐targeted therapies to combat metastatic adenocarcinoma.

## Author Contributions

**Yang Sun:** conceptualization (lead), funding acquisition (lead), project administration (lead), writing – review and editing (lead). **Yang Tian:** conceptualization (equal), data curation (lead), funding acquisition (supporting), investigation (equal), methodology (equal), writing – original draft (lead). **Wei Li:** investigation (equal), methodology (equal), validation (equal). **Qing Zhai:** investigation (supporting), methodology (supporting), validation (equal). **Ying Yu:** investigation (equal), methodology (equal), validation (equal). **Jiaxin Yuan:** investigation (supporting), methodology (supporting). **Yan Ma:** investigation (supporting), methodology (supporting). **Jingjing Yang:** investigation (supporting). **Mingyue Li:** investigation (supporting). **Wenwen Chang:** investigation (supporting). **Wenjing Li:** methodology (supporting). **Keke Huang:** investigation (supporting). **Chongran Sun:** investigation (supporting). **Chen Zeng:** investigation (supporting), methodology (supporting). **Yingdi Sun:** investigation (supporting), methodology (supporting). **Jiabao Gu:** investigation (supporting), methodology (supporting). **Huilin Zhang:** investigation (supporting). **Dameng Li:** methodology (supporting). **Yanan Yu:** methodology (supporting). **Lu Hu:** methodology (supporting), supervision (supporting). **Peng Zhang:** methodology (supporting). **Bo Ma:** supervision (supporting). **Junnian Zheng:** funding acquisition (equal), project administration (equal). **Pan Li:** funding acquisition (equal), investigation (equal), methodology (supporting), supervision (supporting), writing – review and editing (supporting). **Feng Guo:** funding acquisition (equal), methodology (supporting), supervision (equal).

## Conflicts of Interest

The authors declare no conflicts of interest.

## Supporting information


**Figure S1:** Analysis of DHHC family members' mutation, amplification and expression levels across various cancer types. (A) Waterfall plot of mutation analysis of DHHC family members across pan‐cancer samples. (B) mutation table shows the mutation cases of DHHC family members across different cancer types. (C) plot displays the amplification and deletion events of DHHC family members across pan‐cancer samples. (D) Copy number variation (CNV) analysis of DHHC family members across pan‐cancer samples. (E) Expression analysis of DHHC family members across different cancer types.

**Figure S2:** DHHC9‐mediated migration depends on palmitoylation. (A) The structural variant, amplified, mutated and mRNA level of DHHC9 in various adenocarcinomas according to the TCGA cohort. (B) qPCR detection of doxycycline‐regulated *DHHC9* knockdown efficiency in HCT116, A549 and DLD1 cells. (C) Cell viability of doxycycline‐induced DHHC9‐knockdown DLD1 cells measured at indicated times. (D) Transwell assay for detecting the influence of doxycycline alone on cell migration within 48 h in HCT116 cells. (E) Transwell migration assay with the 24‐well Transwell system in DLD1 cells and quantitative analysis. Data are mean ± SEM; *p < 0.05, **p < 0.01, ***p < 0.001.

**Figure S3:** Gene ontology enrichment analysis of DHHC9 substrates and Validation of DHHC9 knockdown efficiency. Overrepresented GO terms in biological processes category of DHHC9 substrates identified with modified ABE in HCT116 (A) and A549 (B) cells. Overrepresented GO terms in cellular component category of DHHC9 substrates identified with modified ABE in HCT116 (C) and A549 cells (D). (E) The efficiency of doxycycline‐regulated *DHHC9* knockdown in HEK293T cells. (F) Western blot analysis of STRN4 palmitoylation in doxycycline‐induced *DHHC9* knockdown HEK293T cells with sh*DHHC9* oligo#1 (F) and oligo#2 (G).

**Figure S4:** DHHC9 promotes cell migration through the Hippo‐YAP signalling pathway. (A) Fisher's exact test using RNA‐seq dataset of DHHC9‐knockdown (vs. control) and STRN4‐C701S overexpression (vs. STRN4‐WT) in HCT116 cells. Two‐tailed *p* value and odds ratio are shown. (B) Correlation of YAP/TAZ/TEAD target genes between DHHC9 knockdown (vs. control) and STRN4 palmitoylation deficiency C701 overexpression (vs. STRN4‐WT) in HCT116 cells. *x*‐axis: fold‐change in STRN4‐C701S vs. STRN4‐WT overexpression; *y*‐axis: fold‐change in DHHC9‐knockdown vs. control cells. The line indicates linear regression fit (*p* = 0.0433). (C,D) Knockdown of DHHC9 in DLD‐1 (C) and A549 (D) cells suppressed YAP downstream gene transcription. (E) Western blot analysis of the effect of STRN4‐WT and palmitoylation‐deficient STRN4‐C701S overexpression on YAP phosphorylation in HCT116 cells. (F) Western blot analysis of Flag‐STRN4 and HA‐DHHC9 expression after MGH‐CP1 treatment. (G) Cell viability of HCT116 cells transfected with DHHC9 and STRN4 plasmid after MGH‐CP1 treatment. Data represent the mean ± SEM (*n* = 6), statistical significances were determined by unpaired two‐sided Student's *t*‐test.

**Figure S5:** Development of Small‐Molecule Inhibitors Targeting DHHC9. (A) Compounds with top 10 XPGscore in drug virtual screening. Compounds with red font were choosed for following validation. (B) The docking result of DHHC9 with treprostinil and 10‐HPCT presented by PyMOL software. PDB of DHHC9: 8HF3. (C,D) Effect of Estriol, neobavaisoflavone, 10‐HCPT, Treprostinil (C) and vatosertib on STRN4 palmitoylation. (D) Effect of 10‐HCPT and Treprostinil on DHHC9 palmitoylation. (E) Cell viability of HCT116 cells after Treprostinil (E) and 10‐HCPT (F) treated for indicated time. (G) Transwell assay of 10‐HCPT treated HCT116 cells after Flag‐DHHC9 or Flag‐STRN4 overexpression. (H) Quantitative analysis of the migration area in (G). For (E) and (F), data represent the mean ± SEM, *n* = 6. For (H), data represent the mean ± SEM, *n* = 5. Statistical significances were determined by unpaired two‐sided Student's *t*‐test.

**Figure S6:** Schematic model of the DHHC9–STRN4–YAP axis in tumour metastasis and its pharmacological inhibition. Left: In adenocarcinoma cells, DHHC9 catalyses S‐palmitoylation of STRN4 at cysteine 701, enhancing recruitment of the STRIPAK (PP2A) complex. This facilitates dephosphorylation of YAP, leading to its nuclear translocation, interaction with TEAD transcription factors and activation of metastasis‐related genes.
Right: Pharmacological inhibition of DHHC9 by Treprostinil or 10‐hydroxycamptothecin (10‐HCPT) suppresses STRN4 palmitoylation, thereby impairing YAP dephosphorylation, promoting its cytoplasmic retention and degradation and ultimately blocking YAP‐driven transcriptional programs. The TEAD inhibitor MGH‐CP1 further disrupts YAP–TEAD interactions to inhibit downstream gene expression.

**Table S1:** DHHC9 substrates identified by modified ABE in HCT116 cells.

**Table S2:** DHHC9 substrates identified by modified ABE in A549 cells.

## Data Availability

The RNA‐seq dataset generated for the current study is available in the Sequence Read Archive (SRA) with Accession Number PRJNA1215392. The proteomics data can be accessed with the identifier PXD060045 through the ProteomeXchange Consortium (http://proteomecentral.proteomexchange.org). The remaining data supporting the findings of this study are available within the article and its supplemental documents. Any additional information required will be available upon request from the lead contact.
