# Supplementary figures and images for: Pharmacological Targeting of DHHC9‐Mediated STRN4 Palmitoylation to Suppress YAP‐Driven Cancer Metastasis
Source: J Cell Mol Med. 2025 Sep 3;29(17):e70815. doi: 10.1111/jcmm.70815 (PMC12408345; doi:10.1111/jcmm.70815)

## Supplementary Figure 1

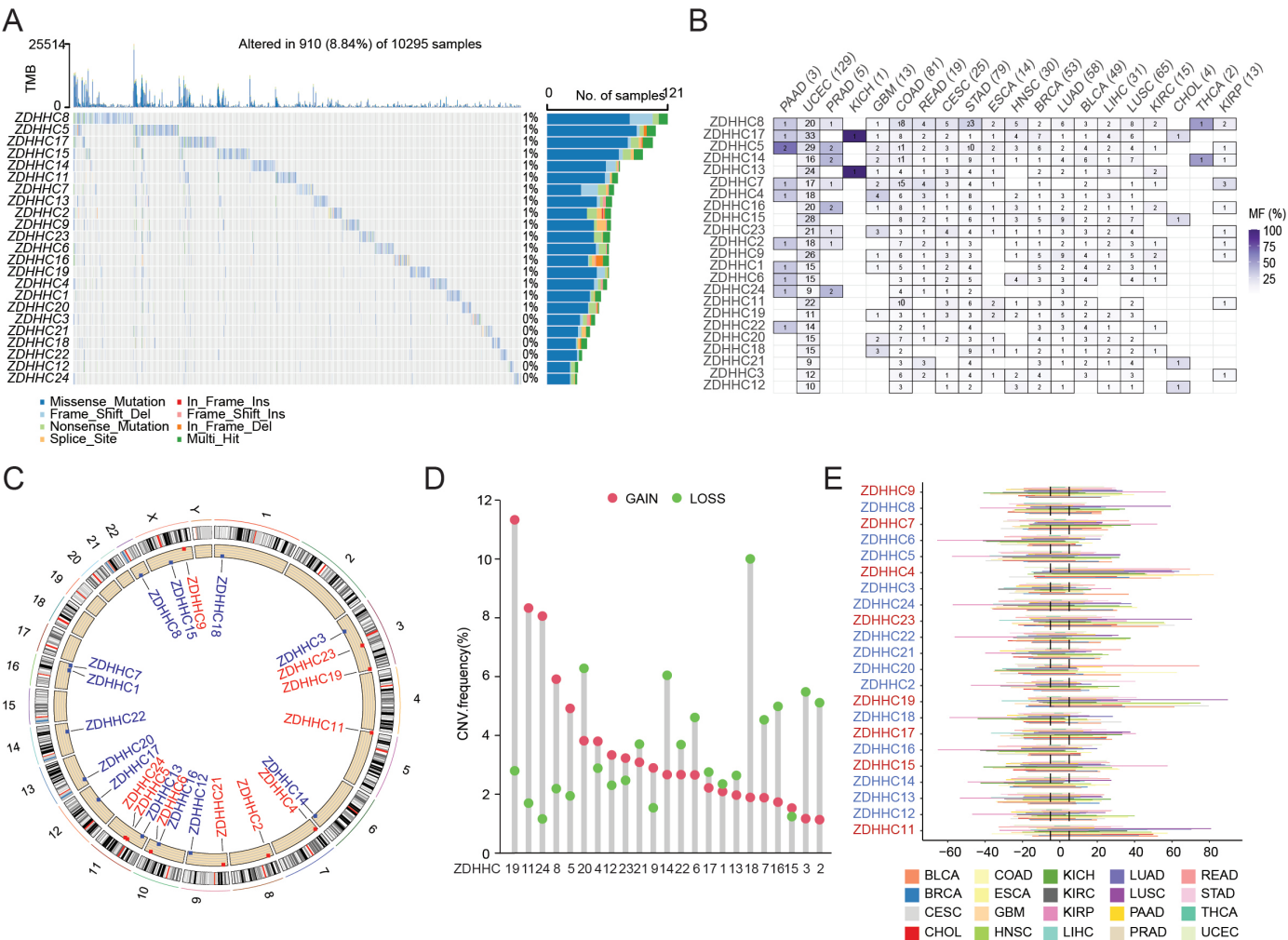

Supplement: Supplementary file 1 — Figure S1: Analysis of DHHC family members' mutation, amplification and expression levels across various cancer types. (A) Waterfall plot of mutation analysis of DHHC family members across pan‐cancer samples. (B) mutation table shows the mutation cases of DHHC family members across different cancer types. (C) plot displays the amplification and deletion events of DHHC family members across pan‐cancer samples. (D) Copy number variation (CNV) analysis of DHHC family members across pan‐cancer samples. (E) Expression analysis of DHHC family members across different cancer types. [file JCMM-29-e70815-s001.pdf]

Supplementary Figure 2

A

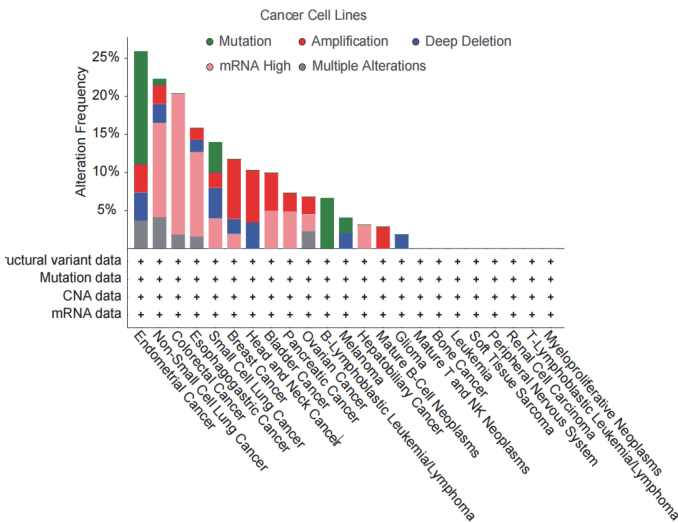

B

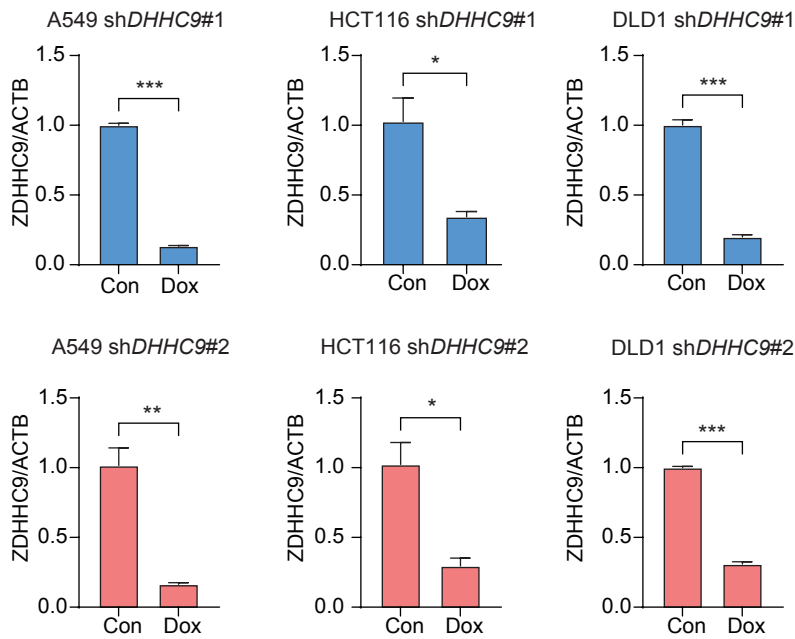

C

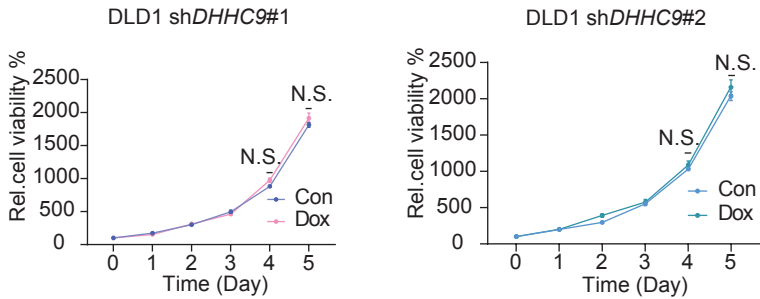

D

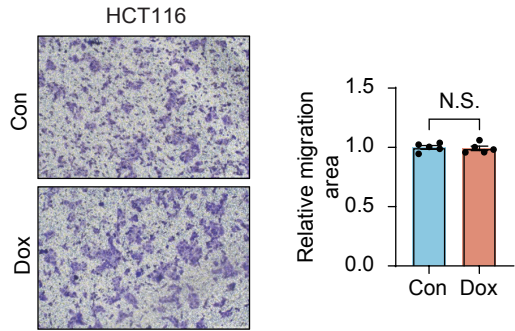

E

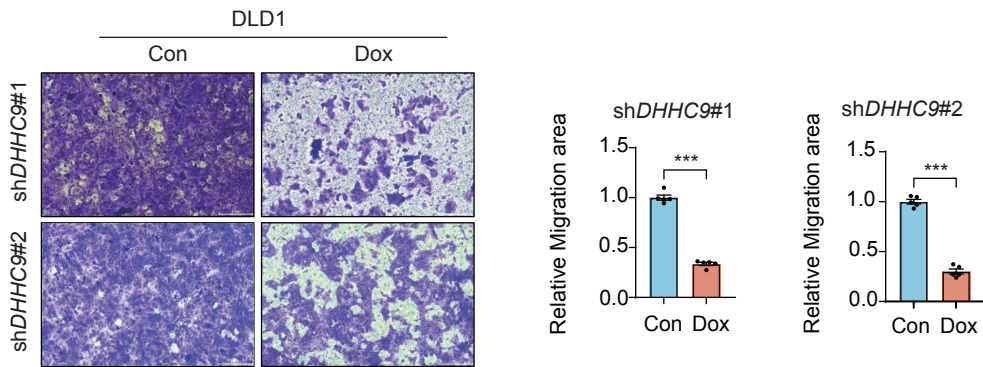

Supplement: Supplementary file 2 — Figure S2: DHHC9‐mediated migration depends on palmitoylation. (A) The structural variant, amplified, mutated and mRNA level of DHHC9 in various adenocarcinomas according to the TCGA cohort. (B) qPCR detection of doxycycline‐regulated DHHC9 knockdown efficiency in HCT116, A549 and DLD1 cells. (C) Cell viability of doxycycline‐induced DHHC9‐knockdown DLD1 cells measured at indicated times. (D) Transwell assay for detecting the influence of doxycycline alone on cell migration within 48 h in HCT116 cells. (E) Transwell migration assay with the 24‐well Transwell system in DLD1 cells and quantitative analysis. Data are mean ± SEM; *p < 0.05, **p < 0.01, ***p < 0.001. [file JCMM-29-e70815-s007.pdf]

Supplementary Figure 3

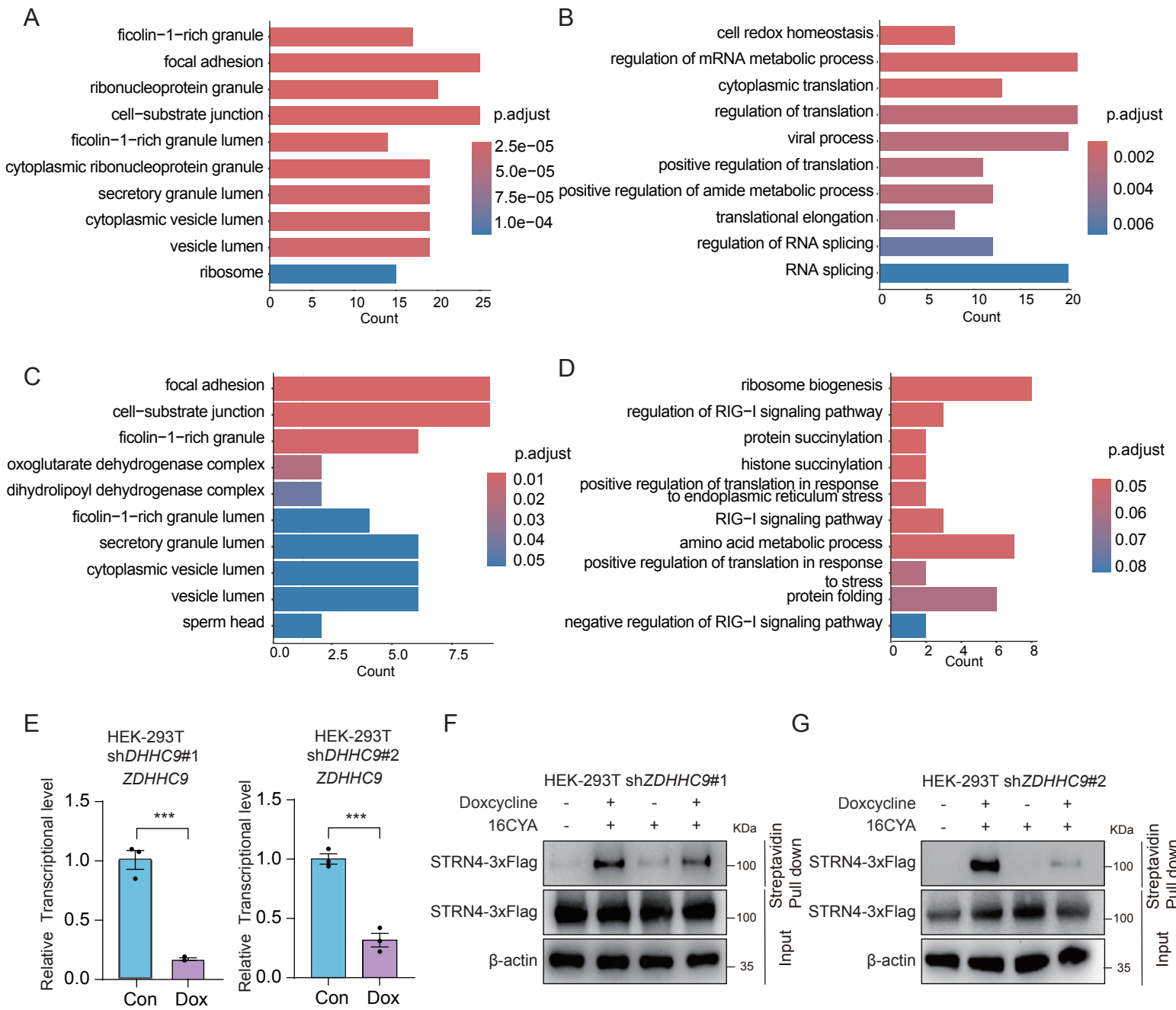

Supplement: Supplementary file 3 — Figure S3: Gene ontology enrichment analysis of DHHC9 substrates and Validation of DHHC9 knockdown efficiency. Overrepresented GO terms in biological processes category of DHHC9 substrates identified with modified ABE in HCT116 (A) and A549 (B) cells. Overrepresented GO terms in cellular component category of DHHC9 substrates identified with modified ABE in HCT116 (C) and A549 cells (D). (E) The efficiency of doxycycline‐regulated DHHC9 knockdown in HEK293T cells. (F) Western blot analysis of STRN4 palmitoylation in doxycycline‐induced DHHC9 knockdown HEK293T cells with shDHHC9 oligo#1 (F) and oligo#2 (G). [file JCMM-29-e70815-s005.pdf]

Supplementary Figure 4

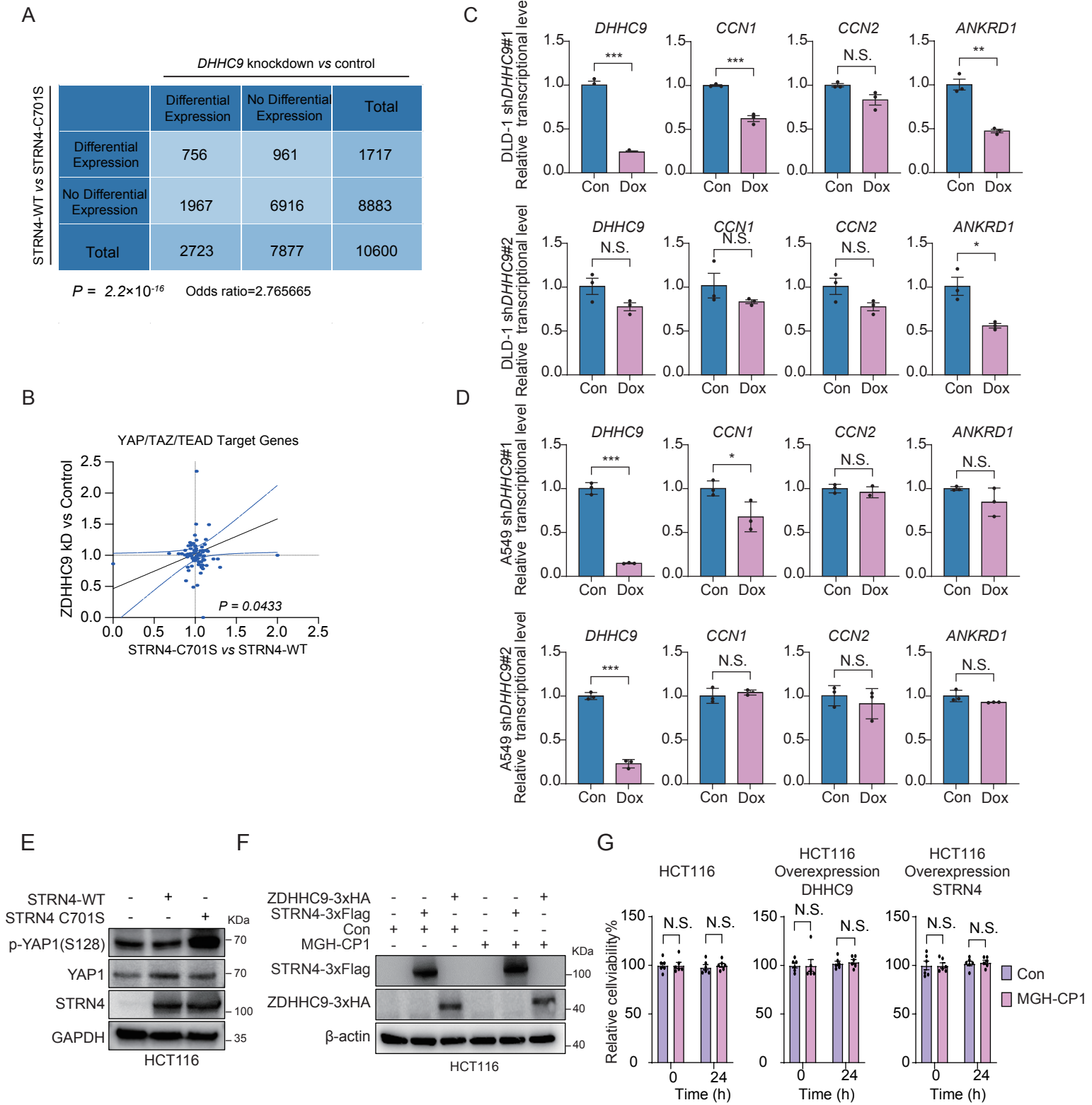

Supplement: Supplementary file 4 — Figure S4: DHHC9 promotes cell migration through the Hippo‐YAP signalling pathway. (A) Fisher's exact test using RNA‐seq dataset of DHHC9‐knockdown (vs. control) and STRN4‐C701S overexpression (vs. STRN4‐WT) in HCT116 cells. Two‐tailed p value and odds ratio are shown. (B) Correlation of YAP/TAZ/TEAD target genes between DHHC9 knockdown (vs. control) and STRN4 palmitoylation deficiency C701 overexpression (vs. STRN4‐WT) in HCT116 cells. x‐axis: fold‐change in STRN4‐C701S vs. STRN4‐WT overexpression; y‐axis: fold‐change in DHHC9‐knockdown vs. control cells. The line indicates linear regression fit (p = 0.0433). (C,D) Knockdown of DHHC9 in DLD‐1 (C) and A549 (D) cells suppressed YAP downstream gene transcription. (E) Western blot analysis of the effect of STRN4‐WT and palmitoylation‐deficient STRN4‐C701S overexpression on YAP phosphorylation in HCT116 cells. (F) Western blot analysis of Flag‐STRN4 and HA‐DHHC9 expression after MGH‐CP1 treatment. (G) Cell viability of HCT116 cells transfected with DHHC9 and STRN4 plasmid after MGH‐CP1 treatment. Data represent the mean ± SEM (n = 6), statistical significances were determined by unpaired two‐sided Student's t‐test. [file JCMM-29-e70815-s002.pdf]

Supplementary Figure 6

Tumor Metastasis

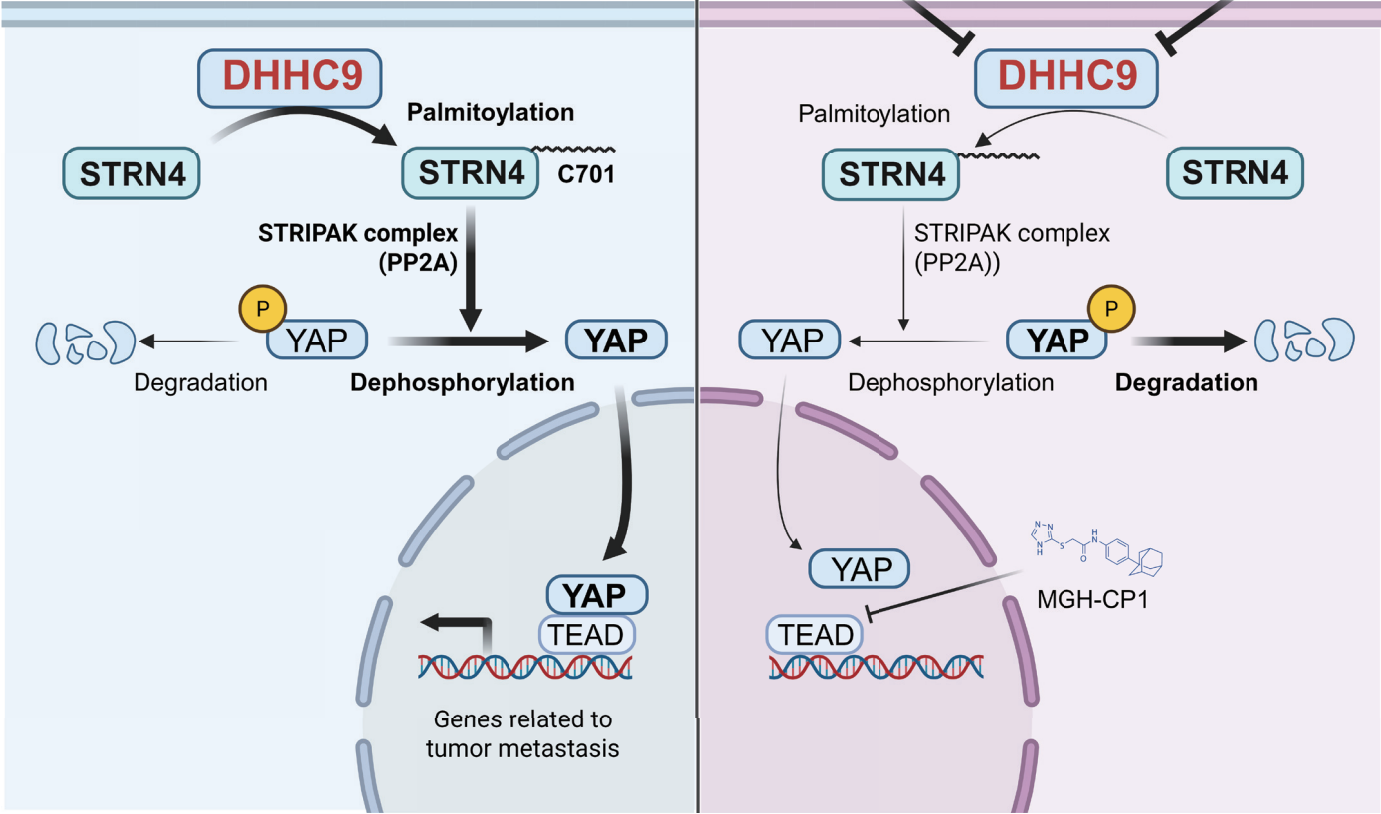

Supplement: Supplementary file 6 — Figure S6: Schematic model of the DHHC9–STRN4–YAP axis in tumour metastasis and its pharmacological inhibition. Left: In adenocarcinoma cells, DHHC9 catalyses S‐palmitoylation of STRN4 at cysteine 701, enhancing recruitment of the STRIPAK (PP2A) complex. This facilitates dephosphorylation of YAP, leading to its nuclear translocation, interaction with TEAD transcription factors and activation of metastasis‐related genes. Right: Pharmacological inhibition of DHHC9 by Treprostinil or 10‐hydroxycamptothecin (10‐HCPT) suppresses STRN4 palmitoylation, thereby impairing YAP dephosphorylation, promoting its cytoplasmic retention and degradation and ultimately blocking YAP‐driven transcriptional programs. The TEAD inhibitor MGH‐CP1 further disrupts YAP–TEAD interactions to inhibit downstream gene expression. [file JCMM-29-e70815-s004.pdf]
