# Supplementary material for: Pharmacological Targeting of DHHC9‐Mediated STRN4 Palmitoylation to Suppress YAP‐Driven Cancer Metastasis
Source: J Cell Mol Med. 2025 Sep 3;29(17):e70815. doi: 10.1111/jcmm.70815 (PMC12408345; doi:10.1111/jcmm.70815)

Supplementary Figure 5

A

PubChem ID	Compound Name	RuleOfFive	XP GScore	MM-GBSA dG Bind(kcal/mol)
21148065	6,8-Diprenylorobol	0	-10.561	-57.42
13347321	AKOS022184657	0	-10.52	-59.6
6918140	Treprostinil	0	-10.501	-60.75
637394	Hirsutenone	0	-10.201	-52.47
619402	Przewaquinone A	0	-9.909	-58.71
5756	Estriol	0	-9.55	-47.12
54766013	Vactosertib	0	-9.549	-66.33
97226	10-Hydroxycamptothecin	0	-9.237	-46.76
5320053	Neobavaisoflavone	0	-9.069	-51.8
122224470	Isoleojaponin	0	-9.568	-46.92

B

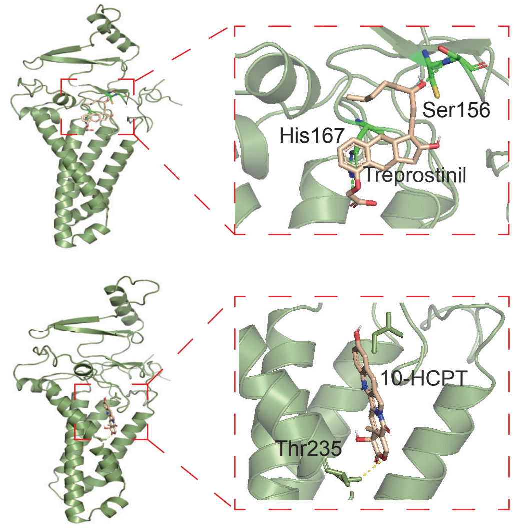

C

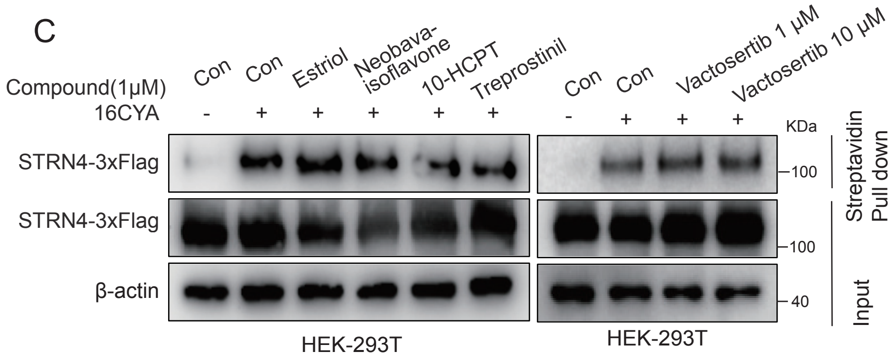

D

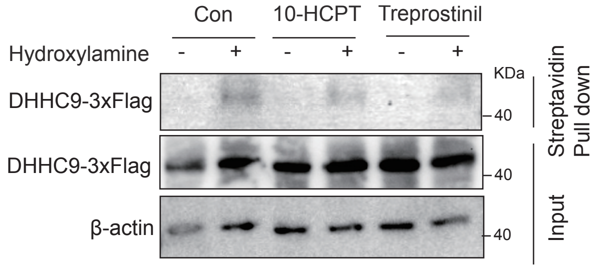

E

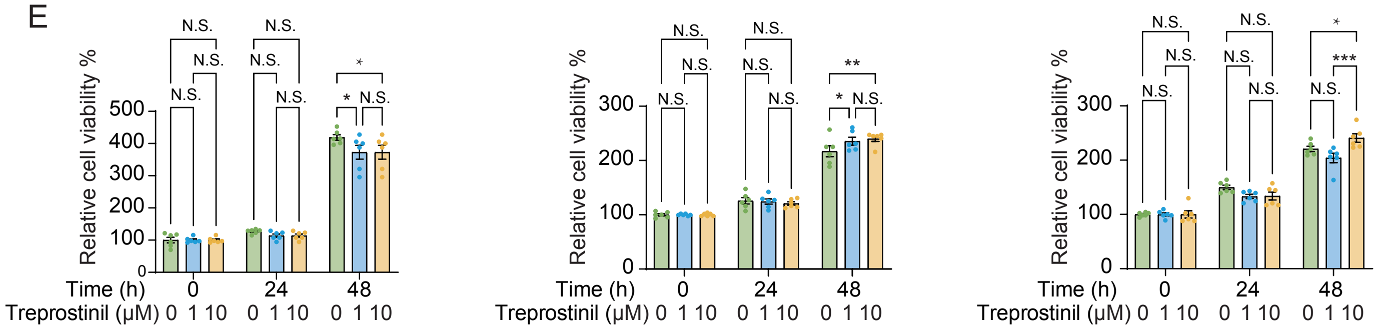

F

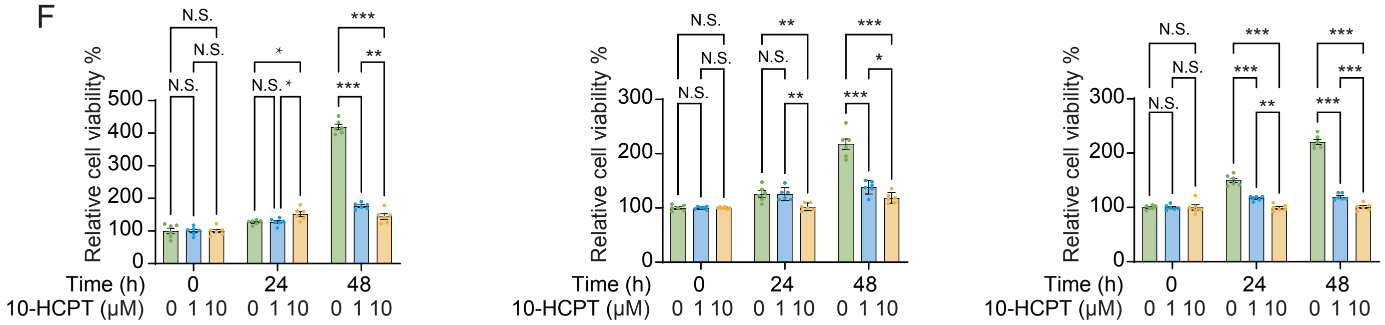

G

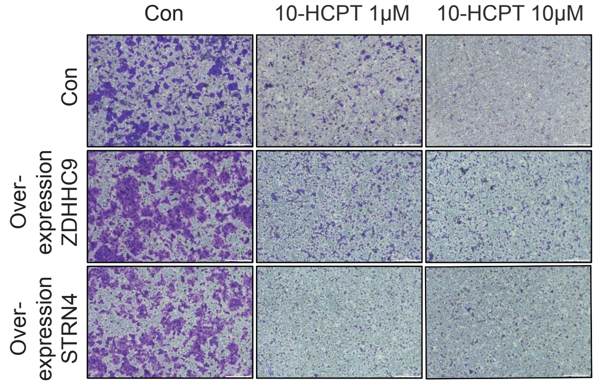

H

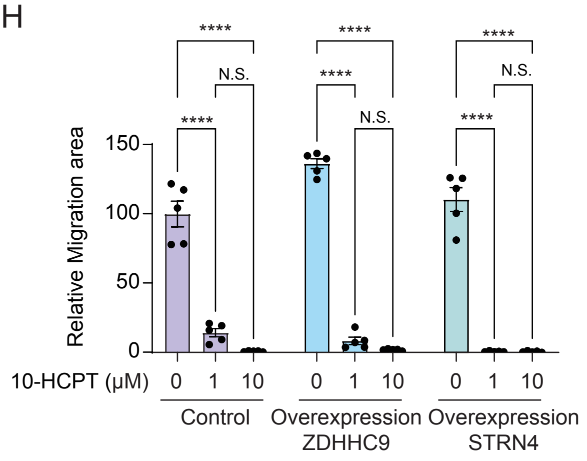

Supplement: Supplementary file 5 — Figure S5: Development of Small‐Molecule Inhibitors Targeting DHHC9. (A) Compounds with top 10 XPGscore in drug virtual screening. Compounds with red font were choosed for following validation. (B) The docking result of DHHC9 with treprostinil and 10‐HPCT presented by PyMOL software. PDB of DHHC9: 8HF3. (C,D) Effect of Estriol, neobavaisoflavone, 10‐HCPT, Treprostinil (C) and vatosertib on STRN4 palmitoylation. (D) Effect of 10‐HCPT and Treprostinil on DHHC9 palmitoylation. (E) Cell viability of HCT116 cells after Treprostinil (E) and 10‐HCPT (F) treated for indicated time. (G) Transwell assay of 10‐HCPT treated HCT116 cells after Flag‐DHHC9 or Flag‐STRN4 overexpression. (H) Quantitative analysis of the migration area in (G). For (E) and (F), data represent the mean ± SEM, n = 6. For (H), data represent the mean ± SEM, n = 5. Statistical significances were determined by unpaired two‐sided Student's t‐test. [file JCMM-29-e70815-s006.pdf]
